# ADP-ribosyltransferase-based biocatalysis of nonhydrolyzable NAD+ analogs

**DOI:** 10.1016/j.jbc.2024.108106

**Published:** 2024-12-18

**Authors:** Moona Sakari, Rajendra Bhadane, Sujit Kumar, Rita Azevedo, Morteza Malakoutikhah, Ahmadreza Masoumi, Dene R. Littler, Harri Härmä, Kari Kopra, Arto T. Pulliainen

**Affiliations:** 1 Institute of Biomedicine, University of Turku, Turku, Finland; 2 Department of Chemistry, University of Turku, Turku, Finland; 3 Infection and Immunity Program & Department of Biochemistry and Molecular Biology, Biomedicine Discovery Institute, Monash University, Clayton, Victoria, Australia

**Keywords:** ADP-ribosyltransferase, NAD+, enzyme promiscuity, biocatalysis, nucleotide analog

## Abstract

Enzyme promiscuity is the ability of an enzyme to catalyze an unexpected side reaction in addition to its main reaction. Here, we describe a biocatalytic process to produce nonhydrolyzable NAD+ analogs based on the ADP-ribosyltransferase activity of pertussis toxin PtxS1 subunit. First, in identical manner to normal catalysis, PtxS1 activates NAD+ to form the reactive oxocarbenium cation. Subsequently, the electrophilic ribose 1′ carbon of the oxocarbenium cation is subject of an attack by the nitrogen atom of an amino group coupled to nicotinamide mimicking compounds. The nitrogen atom acts as the nucleophile instead of the natural sulfur atom substrate of the human Gαi protein. The invention builds on structural data indicating the presence of an NAD+ analog, benzamide amino adenine dinucleotide, at the NAD+ binding site of PtxS1. This was witnessed upon cocrystallization of PtxS1 with NAD+ and 3-aminobenzamide (3-AB). A pharmacophore-based screening on 3-AB followed by quantum mechanical simulations identified analogs of 3-AB with capacity to react with the oxocarbenium cation. Based on HPLC and mass spectrometry, we confirmed the formation of benzamide amino adenine dinucleotide by PtxS1, and also identified two new chemical entities. We name the new entities as isoindolone amine adenine dinucleotide, and isoquinolinone amine adenine dinucleotide, the latter being a highly fluorescent compound. The new NAD+ analogs emerge as valuable tools to study the structural biology and enzymology of NAD+ binding and consuming enzymes, such as human poly(ADP-ribose) polymerases and bacterial ADP-ribosyltransferase exotoxins, and to advance the ongoing drug development efforts.

Nicotinamide adenine dinucleotide (NAD+) and its reduced form NADH are key coenzymes in the metabolic redox reactions, *i.e.*, in glycolysis, tricarboxylic acid cycle, and oxidative phosphorylation. Hundreds of other enzymatic reactions also rely on these dinucleotides. Notably, NAD+ is functionally essential for a large group of non-redox NAD+ cleaving enzymes, including poly(ADP-ribose) polymerases (PARPs), which are important molecular players, for example, in the maintenance and repair of DNA to safeguard genomic stability and epigenetic regulation and chromatin remodeling (1). There is a high catalytic variability with the non-redox NAD+ cleaving enzymes. For example, nicotinamide is released from NAD+ and the rest of the molecule is either released into solution as ADP-ribose or acetyl-ADP-ribose, covalently attached on target macromolecules, mostly on proteins as mono- and poly-ADP-ribose chains, or is subject of cyclization reaction to form cyclic-ADP-ribose (1). In addition to the mammalian NAD+ cleaving enzymes, there are a number of bacterial exotoxins that in the host cell cytosol either covalently modify host cell proteins *via* mono-ADP-ribosylation, *e.g.*, pertussis toxin (2), or deplete NAD+ *via* concomitant nicotinamide and ADP-ribose release, *e.g.*, tuberculosis necrotizing toxin (3). Some bacterial NAD+ cleaving enzymes act inside the bacterium. For example, the MbcT toxin of the MbcTA toxin-antitoxin system of *Mycobacterium tuberculosis* depletes NAD+ *via* concomitant release of nicotinamide and phosphate-ADP-ribose (4). It is characteristic of the NAD+ cleaving enzymes that they release nicotinamide but the second reaction product, *i.e.*, soluble or covalently conjugated to macromolecules, varies with the specific enzymatic activity.

The NAD+ cleaving nonredox enzymes are potent drug targets. The drug developmental pipelines have focused mostly on NAD+ binding site targeting molecules, which mimic NAD+ or parts of it (5). Most notably, due to the central role of PARP1 in the DNA damage response, small-molecule drugs interfering with NAD+ binding, *e.g.*, olaparib and rucaparib, have entered clinical use in cancer treatments, *e.g.*, in ovarian cancer, in particular in patients with homologous recombination defects due to BRCA1/BRCA2 mutations (6, 7, 8). In addition, multiple preclinical development pipelines of pathogen-specific antibacterial small molecular weight drugs are focused on bacterial ADP-ribosyltransferase (ART) exotoxins (9), *e.g.*, to therapeutically or to prophylactically inhibit pertussis toxin (2, 10).

The NAD+ cleaving enzyme-targeted drug development is challenging due to the high abundancy of homologous and nonhomologous NAD+ binding proteins. For example, several PARP1 inhibitors inhibit the activity of other PARPs, such as PARP2 and PARP3 (5). Thus, more detailed understanding of the NAD+ binding modes would be useful to improve the fit and specificity of inhibitors. To this end, catalytically compromised mutants (10, 11), wt forms with NAD+ under catalytically nonpermissive conditions, *e.g.*, under cryogenic flash freezing (12) or under incubation with catalysis-inhibiting salt ion (10), or wt forms with NAD+ analogs that have diminished or neglectable nicotinamide displacement, *i.e.*, carba-NAD+ (13, 14), thioribose-NAD+ (15) and benzamide adenine dinucleotide (BAD) (16, 17), have been used in crystallization studies. Carba-NAD+ and thioribose-NAD+ are synthetic compounds identical to NAD+ except for one substitution, where an oxygen atom adjacent to the anomeric linkage bearing nicotinamide is replaced with a methylene group (carba-NAD+) or sulfur atom (thioribose-NAD+). These modifications make carba-NAD+ and thioribose-NAD+ inert in nicotinamide displacement reactions, although they bind to NAD+ consuming enzymes in a manner similar to NAD+. Carba-NAD+ has been used, for example, to obtain NAD+ binding mode information of sirtuins, SIRT3, and SIRT5 (14). BAD is another nonhydrolyzable NAD+ analog where the nitrogen atom of the nicotinamide ring is replaced with a carbon atom converting the benzamide moiety into a poor leaving group (16, 17). The BAD has, for example, been used to obtain NAD+ binding mode information of PARP1 in order to understand how DNA damage leads to the activation of PARP1 (18).

The nonhydrolyzable NAD+ analogs carba-NAD+ and BAD are commercially available, but expensive to purchase in large quantities, and not widely used, despite their clear benefits in structural elucidation of NAD+ binding modes to proteins. Recently, we detected the binding of a novel chemical entity, which we named benzamide amino adenine dinucleotide (BaAD) at the NAD+ binding site of pertussis toxin (10). The binding pose was acquired after the crystallization of a Cys41Ser-mutant of the truncated ART subunit PtxS1 (pertussis toxin S1 subunit) together with NAD+ and 3-aminobenzamide, which is a weak millimolar inhibitor of PtxS1 (2, 10). Here, we set out to confirm the formation of BaAD by PtxS1, to investigate the structural and mechanistic basis of its formation, and to assess the feasibility of PtxS1-mediated biocatalytic process to produce BaAD for downstream applications.

## Results

### Analysis of the binding pose of BaAD in PtxS1

**Figure 1:**
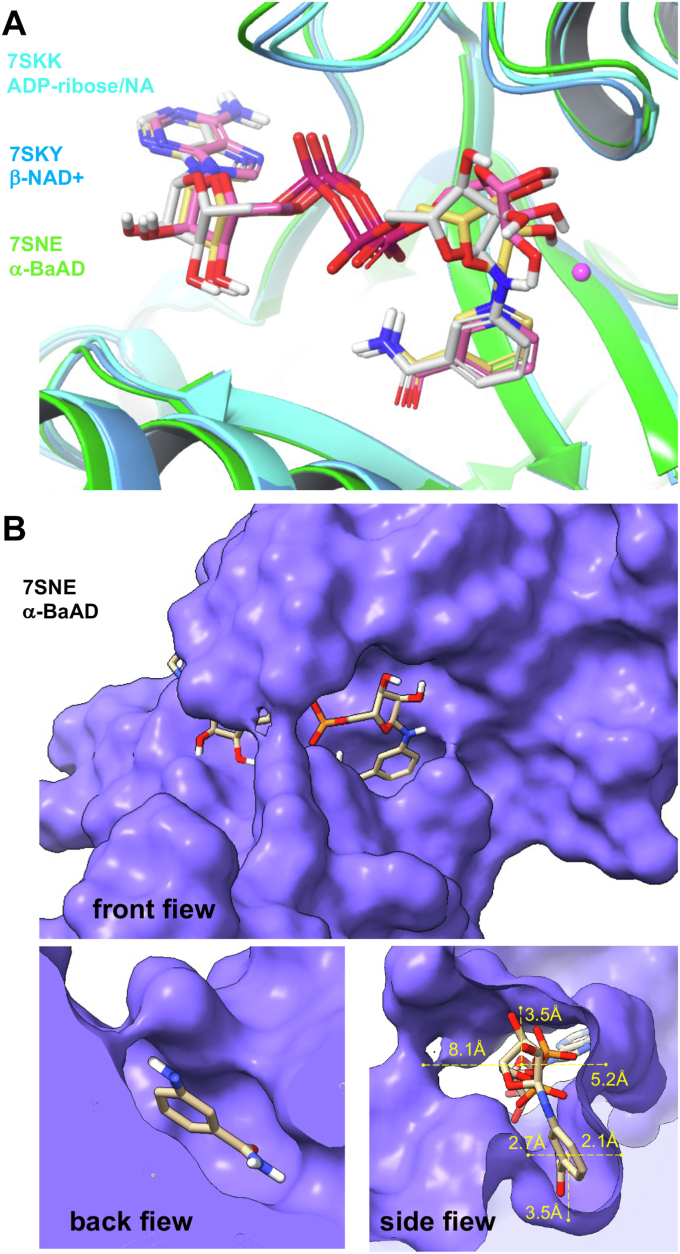
**Binding pose of BaAD in PtxS1.***A*, the cartoon representation of structures of PtxS1 complexed with β-NAD+ (7SKY and β-NAD+ in *orange*), α-BaAD (7SNE and α-BaAD in *gray*) and post NAD+ hydrolysis products ADP-ribose and nicotinamide (NA) (7SKK, ADP-ribose, and nicotinamide in *pink*). The iodide ion of the NAD+ complexed structure is shown as *pink ball*. *B*, the surface representation of PtxS1 showing location of the NAD+ binding pocket and the cocrystallized structure of α-BaAD in *front*, *back*, and *side* views. *Back* view—the α-BaAD and protein surface is clipped to show the nicotinamide binding pocket and orientation of the 3-AB remnant. *Side* view—the figure depicts the structural arrangement of α-BaAD, emphasizing the spatial separations between the ribose ring and the adjacent surface, as well as between the phenyl ring and the neighboring surface. The phenyl ring is firmly ensconced within a hydrophobic pocket created by proximal amino acid residues, whereas the ribose ring has noticeably more distance from the neighboring surface. BaAD, benzamide amino adenine dinucleotide; PtxS1, pertussis toxin S1 subunit.

**Figure 2:**
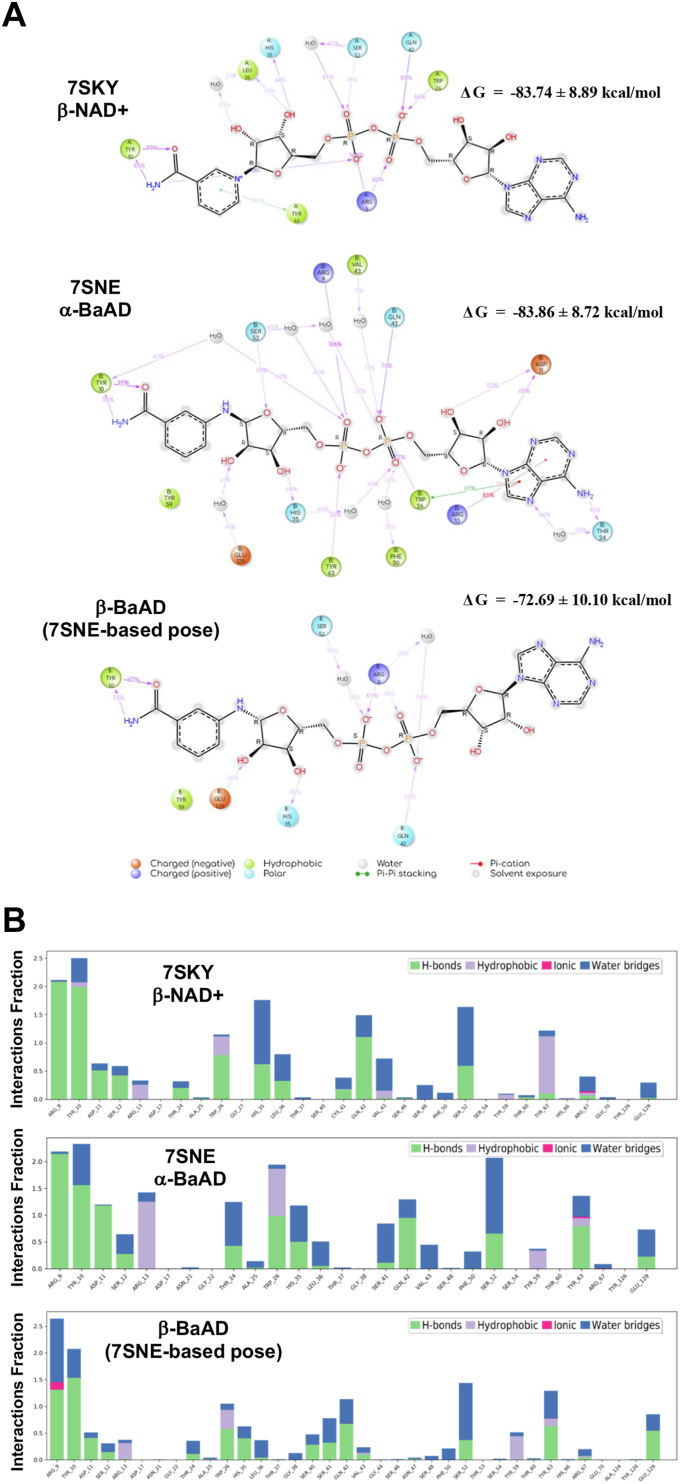
**Modeling of nucleotide binding to PtxS1.***A*, ligand-receptor interaction diagrams showing interactions of PtxS1 with cocrstallized *β*-NAD+ (PDB 7SKY), α-BaAD (PDB 7SNE), and docked *β*-BaAD (based on 7SNE α-BaAD pose) over the course of a 500-ns long molecular dynamics (MD) simulation (% denotes the duration of an interaction from the total simulation time). Only interactions that lasted for more than 30% of the simulation time are considered. *B*, the histogram showing interactions of PtxS1 with cocrystallised *β*-NAD+ (PDB 7SKY), α-BaAD (PDB 7SNE), and docked *β*-BaAD (PDB 7SNE). The stacked bar charts are normalized over the course of a 500-ns MD simulation trajectory. Therefore, the interactions fraction value (*Y*-axis) denotes how long the interaction lasted with respect to the length of the total simulation. If the interactions fraction exceeds 1.0 (100% of the simulation time), the respective residue forms more than one interaction with the ligand. PDB, Protein Data Bank; BaAD, benzamide amino adenine dinucleotide; MD, molecular dynamics; PtxS1, pertussis toxin S1 subunit.

The glycosidic bond between the anomeric 1′ carbon, and the nitrogen of 3-aminobenzamide (3-AB) remnant in BaAD is in alpha configuration (10). Despite this stereochemical difference to β-NAD+, the binding pose of α-BaAD (Protein Data Bank (PDB) 7SNE) in PtxS1 is remarkably similar with the binding pose of β-NAD+ (PDB 7SKY) (Fig. 1*A*). The adenine end, phosphate groups, and the nicotinamide end (β-NAD+) or the 3-AB remnant (α-BaAD) are positioned similarly. This is also evident when α-BaAD binding pose is compared with post β-NAD+ hydrolysis structure with bound α-ADP-ribose and nicotinamide (PDB 7SKK) (Fig. 1*A*). There is a tight fit of the nicotinamide end (β-NAD+) or the 3-AB remnant (α-BaAD) in the catalytic pocket (Fig. 1*B*). Accordingly, binding of the BaAD 3-AB remnant, which in comparison to β-NAD+ nicotinamide has one nitrogen extension, is associated with significant movement of the ribose group (Fig. 1*A*). In order to gain further insights into the binding properties of α-BaAD, we performed docking and binding free energy analysis based on the cocrystallized poses of β-NAD+ and α-BaAD (PDB 7SKY and 7SNE, respectively, Table 1). The molecular dynamics (MD) simulations were also conducted, and, subsequently, binding free energy values were calculated and averaged over the simulation time. Upon comparing the modeled interaction patterns, it became evident that α-BaAD exhibited similar kinds of interactions with PtxS1 as compared with β-NAD+ (Fig. 2). In contrast, the interaction pattern of a hypothetical stereoisomer β-BaAD was notably distinct and displayed weaker interactions with PtxS1. Moreover, the binding free energy values of α-BaAD (Table 1) were more favorable as compared with the β-NAD+ hydrolysis intermediate oxocarbenium cation and the β-NAD+ hydrolysis product α-ADP-ribose. The modeling data imply that α-BaAD has affinity to the NAD+ binding site of PtxS1 where it has a binding pose similar to β-NAD+.

**Table 1:** Modeling of nucleotide binding to PtxS1

Nucleotide	Docking score			Binding free energy by MM-GBSA (ΔG, kcal/mol)		
	7SKY^a^ β-NAD+	7SNE^b^ α-BaAD	1PRT^a^ apo	7SKY^a^ β-NAD+	7SNE^b^ α-BaAD	1PRT^a^ apo
β-NAD+	NA^c^	−15.99	−10.90	−86.59^d^	−94.73	−62.31
β-BaAD	−13.14	−15.64	−12.83	−69.14	−88.21	−75.00
α-BaAD	−8.07	NA^c^	−8.35	−78.60	−97.75^d^	−63.68
β-BAD	−14.33	−18.58	−10.92	−88.64	−80.03	−55.68
β-carba-NAD+	−10.44	−18.58	−9.56	−72.98	−92.90	−91.14
oxocarbenium	−10.93	−12.09	−11.41	−63.98	−53.23	−69.11
α-ADP-ribose	−10.23	−14.22	−11.87	−58.81	−79.49	−69.07

Docking scores and Prime/MM-GBSA binding free energy (ΔG bind) values of the cocrystallized and docked nucleotides at the apo and β-NAD+ -bound as well as α-BaAD-bound protein structures of PtxS1. The natural β-NAD+ substrate, the β-NAD+ hydrolysis intermediate oxocarbenium cation, the β-NAD+ hydrolysis product α-ADP-ribose, and two non-hydrolyzable NAD+ analogs (β-BAD, β-carba-NAD+) were included in the analysis. The β-BaAD is a derivative of β-NAD+ theoretically getting formed upon PtxS1, β-NAD+, and 3-AB coincubation.

a Analysis with NAD+ and analogs based on experimental β-NAD+ cocrystal pose (7SKY).

b Analysis with NAD+ and analogs based on experimental α-BaAD cocrystal pose (7SNE).

c NA, not applicable.

d From experimental cocrystal pose.

### Molecular modeling on the effect of NAD+ binding site mutations to PtxS1

**Figure 3:**
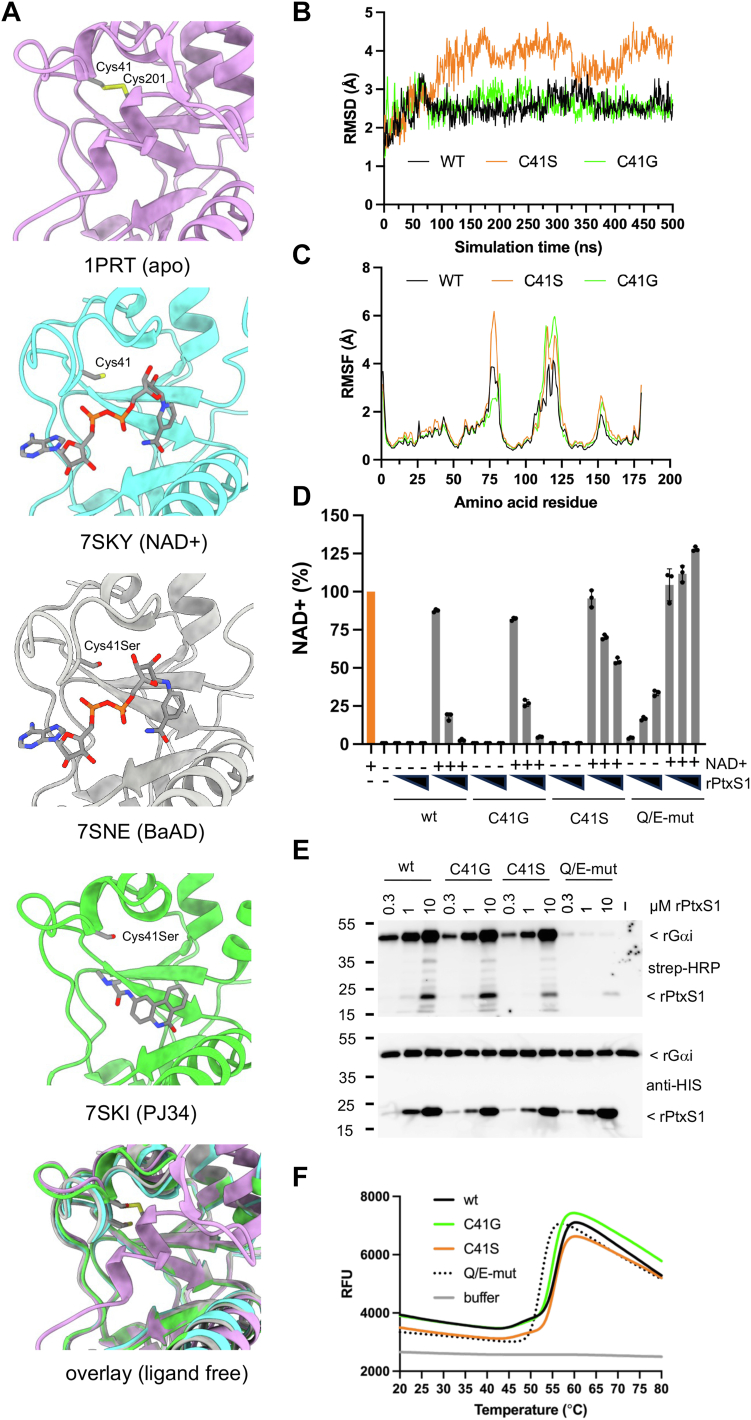
***In silico* and biochemical characterization of a series of PtxS1 mutants.***A*, location of Cys41 in the flexible NAD+ loop of rPtxS1. This loop folds over NAD+, and BaAD, but not over smaller compound PJ34, upon ligand binding to the active site. In the apo-form, Cys41 forms a disulfide bond with Cys201 that locates an autoinhibitory C terminus into the active site until the pertussis toxin gains access to the reducing cytosol. Apo structure of PtxS1 (PDB 1PRT) in *pink*; NAD+ structure of rPtxS1 (PDB 7SKY) in *cyan*; BaAD structure of rPtxS1 (PDB 7SNE) in *gray*; PJ34 structure of rPtxS1 (PDB 7SKI) in *green*. The hydrogens atoms are displayed in the α-BaAD structure (PDB 7SNE) due to its high 1 Å resolution. *B*, the RMSD of the wt, and *in silico* C41S and C41G mutant structures of rPtxS1 with cocrystallized β-NAD+ (NAD+ structure of rPtxS1, PDB 7SKY) during a 500-ns long MD simulation. *C*, the root mean square fluctuation (RMSF) of the residues in the wt and *in silico* C41S and C41G mutant structures of rPtxS1 with cocrystallized β-NAD+ (NAD+ structure of rPtxS1, PDB 7SKY) during a 500-ns long molecular dynamics (MD) simulation (active site NAD+ loop, 38–49; H3-helix residues, 56–80; flexible loop region, 112–127; flexible loop between *β*8-*β*9, 150–155). *D*, *in vitro* NAD+ consumption activity of rPtxS1 as measured with a 96-well fluorometric assay. NAD+ is chemically converted at the end of the 60 min consumption assay into a fluorescent molecule. Values are given as mean ± SD percentages of the NAD+ control (n = 3, one representative experiment). IMAC-purified forms of rPtxS1 were analyzed at concentrations of 1, 5, and 10 μM, with or without NAD+ addition (10 μM). *E*, *in vitro* ADP-ribosylation assay for IMAC-purified rPtxS1 with biotin-NAD+, and SEC-purified substrate protein rGαi (1 μM). All the proteins are HIS-tagged. Protein-conjugated ADP-ribose-biotin was detected with streptavidin-HRP. *F*, DSF curves of IMAC-purified wt and mutant forms rPtxS1 (Tm 54.28 °C, wt; Tm 53.85 °C, Cys41Gly; Tm 54.46 °C, Cys41Ser; Tm 51.48 °C, Q/E-mut). Q/E-mut refers to Q127D/E129D double mutant of rPtxS1. DSF curves are drawn based on mean values of triplicate runs. BaAD, benzamide amino adenine dinucleotide; DSF, differential scanning fluorometry; PtxS1, pertussis toxin S1 subunit; IMAC, immobilized metal affinity chromatography; SEC, size-exclusion chromatography; HRP, horseradish peroxidase.

Previously, we witnessed α-BaAD bound to C41S mutant of truncated PtxS1 (referred to as C41S-rPtxS1) upon crystallization with β-NAD+ and 3-AB (10). This implies somewhat altered biochemical features of this mutant form of rPtxS1. Cys41 is located at the middle of a flexible loop (referred to as the NAD+ loop) that moves from the apo conformation (PDB 1PRT, (19)) on top of NAD+ upon NAD+ binding (Fig. 3*A*) (10). In the apo form, Cys41 forms a disulfide bond with Cys-201 that locates an autoinhibitory C terminus into the NAD+ binding site until PtxS1 gains access to the reducing cytosol (19). To investigate the effect of C41 mutations, we first performed MD simulations on the structure of rPtxS1 cocrystallized with β-NAD+ and two *in silico* mutant structures, C41S and C41G. The RMSD values were compared over a 500-ns MD simulation. The RMSD of the wt and C41G mutant converged early, reaching stability at around 30-ns and remaining consistent throughout the simulation (Fig. 3*B*). In contrast, the C41S mutant exhibited higher RMSD values, indicating increased structural fluctuations. To identify the specific amino acid residues responsible for these fluctuations, the root mean square fluctuation was analyzed. For the C41S mutant, higher fluctuations were observed in the H3-helix residues (amino acids 56–80), followed by the flexible loop region (amino acids 112–127), the loop between β8 and β9 (amino acids 150–155), and the NAD+ binding site loop (amino acids 38–49). In comparison, the root mean square fluctuation of the wt structure remained relatively stable in the flexible loop region, compared to both the C41S and C41G mutants. However, it exhibited less stability than the C41G mutant in the H3-helix loop and NAD+ binding site loop regions (Fig. 3*C*). We also analyzed the specific amino acid interactions with β-NAD+ (Fig. S1). We observed that both the wt and the C41S mutant exhibited direct hydrogen bond and water-mediated interactions between the amino acid residue at the 41 position and β-NAD+. However, these interactions were lost in the C41G mutant. There was also a loss of interactions with H32, L36, and V42 in the C41G mutant, which was compensated by increased interactions with Y8, Q42, S54, Y59, T60, L123, and Y126. Overall, the modeling exercise indicates that catalytic alterations are probable with both PtxS1 mutants, but, in particular, with the C41S mutant of rPtxS1.

### Biochemical analysis of the effect of NAD+ binding site mutations to the catalytic activity of PtxS1

First, we determined how the mutations affected the NADase activity of rPtxS1 in the absence of its substrate protein inhibitory alpha subunit of the heterotrimeric G-protein (Gαi). As shown in Figure 3*D*, the C41G mutant had similar activity as compared to the wt, but the C41S mutant, and, in particular, the Q127D/E129D double mutant, had a compromised NADase activity. Interestingly, the Q127D/E129D double mutant without NAD+ addition appeared to contain NAD+, which is most likely caused by the carry-over of NAD+ bound to the rPtxS1 during protein expression in the *Escherichia coli*. Secondly, we determined how the mutations affected ADP-ribosylation of the Gαi substrate protein of PtxS1 using the biotin-NAD+ approach. As shown in Figure 3*E*, only the Q127D/E129D double mutant had a compromised ART activity, in accordance with the critical postulated role of Q127 and D129 in oxocarbenium cation formation (10, 19). Therefore, the C41S mutation appears to cause differential effect on rPtxS1 activity depending if the Gαi substrate protein is present or not (Fig. 3*D* vs. 3*E*). Importantly, the C41S and C41G mutations had a negligible and the Q127D/E129D mutation only a minor effect on PtxS1 folding as evidenced by differential scanning fluorometry (DSF) (Fig. 3*F*). Taken together, the biochemical data demonstrate that there are catalytic alterations with C41S mutation. These catalytic alterations together with spatial NAD+ binding site alterations could explain why α-BaAD was detected in C41S mutant crystallization experiments with β-NAD+ and 3-AB coincubation (10).

### Detection of NAD+ binding site amino acid auto-ADP-ribosylation in PtxS1

**Figure 4:**
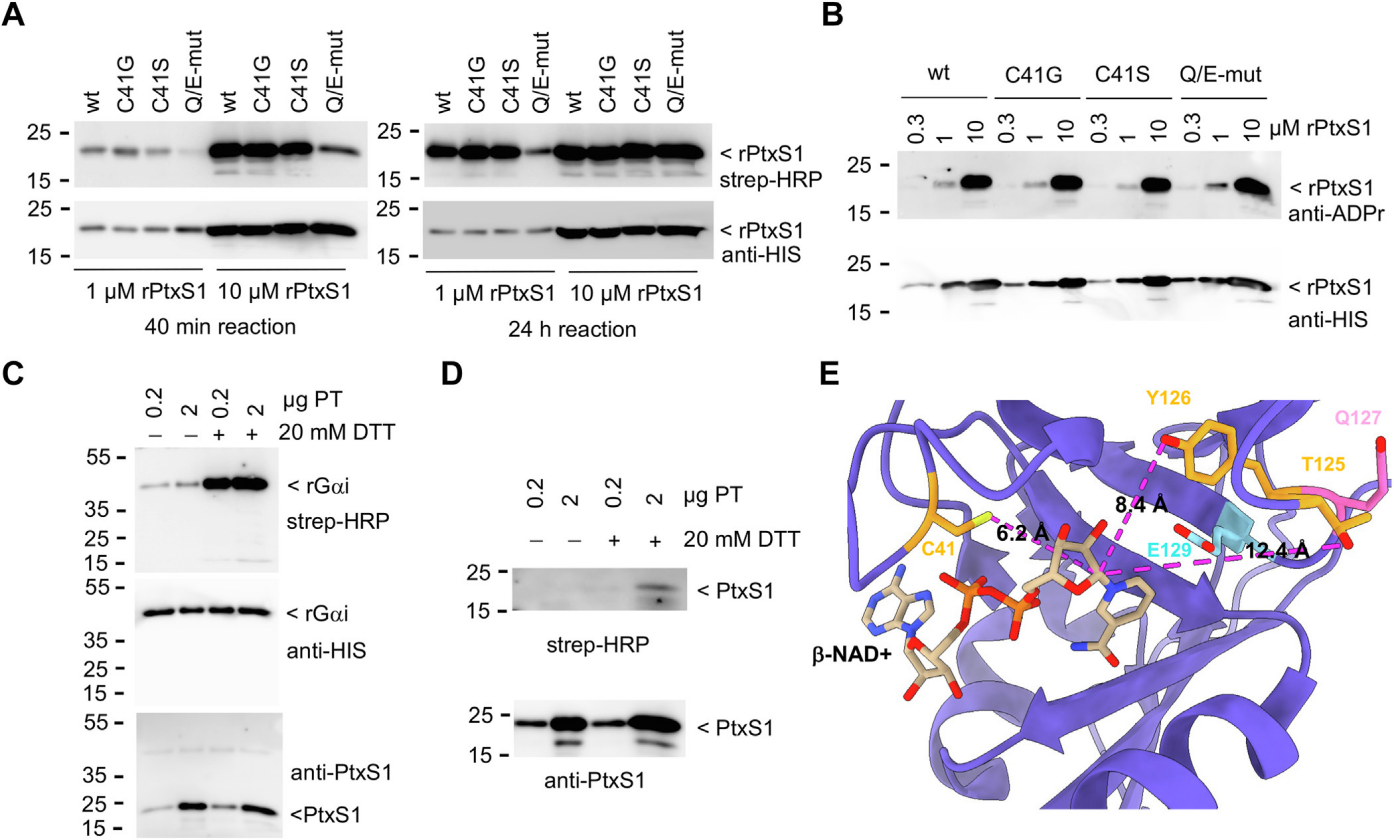
**Auto-ADP-ribosylation activity of PtxS1.***A*, *in vitro* auto-ADP-ribosylation assay for rPtxS1 with biotin-NAD+. Protein-conjugated ADP-ribose-biotin was detected with streptavidin-HRP. Auto-ADP-ribosylation reactions were either incubated for 40 min or 24 h at room temperature before loading into the gel. Q/E-mut refers to Q127D/E129D double mutant of rPtxS1. *B*, detection of ADP-ribose that had been conjugated to rPtxS1 during expression in *E. coli*. Protein-conjugated ADP-ribose was detected with a rabbit monoclonal antibody that recognizes poly- and mono-ADP-ribose. *C*, *in vitro* substrate protein ADP-ribosylation assay for pertussis holotoxin (PT) with biotin-NAD+. The PT was activated by addition of DTT to the reaction mixture, *i.e.*, DTT reduces the disulfide bond that keeps the C-terminal autoinhibitory domain at the catalytic NAD+ binding pocket. Protein-conjugated ADP-ribose-biotin was detected with streptavidin-HRP. *D*, *in vitro* auto-ADP-ribosylation assay for pertussis holotoxin (PT) with biotin-NAD+. The PT was activated by addition of DTT to the reaction mixture. Protein-conjugated ADP-ribose-biotin was detected with streptavidin-HRP. *E*, location of the auto-ADP-ribosylated amino acid residues of rPtxS1. The residues were determined by mass spectrometry (Fig. S2), and are highlighted here in the *β*-NAD+ bound structure of rPtxS1 (PDB 7SKY). The distances from the oxygen (*red*) and sulfur (*yellow*) atoms of the auto-ADP-ribosylated amino acid residues are displayed relative to the location of the anomeric 1′ carbon of *β*-NAD+. PtxS1, pertussis toxin S1 subunit; HRP, horseradish peroxidase; PDB, Protein Data Bank.

We witnessed auto-ADP-ribosylation of rPtxS1 in the biotin-NAD+ experiments, which appeared to be diminished in the Q127D/E129D double mutant of rPtxS1, but not significantly in the other mutants of rPtxS1 (Fig. 3*E*). The auto-ADP-ribosylation activity was also witnessed without the rGαi substrate protein (Fig. 4*A*). Interestingly, when we prolonged the *in vitro* incubation without rGαi from the typical 40 min to 24 h, the Q127D/E129D double mutant appeared like the wt (Fig. 4*A*). Normal level of auto-ADP-ribosylation was also witnessed in the Q127D/E129D double mutant with anti-ADP-ribose Western blotting when we analyzed proteins purified from *Escherichia coli* after overnight expression in auto-induction medium (Fig. 4*B*). This implies that given enough time, as in our biochemical experiments or during an overnight expression in *E. coli*, the catalytically compromised Q127D/E129D double mutant gains a level of auto-ADP-ribosylation indistinguishable from the wt. Auto-ADP-ribosylation was not only detected with our truncated recombinant forms of PtxS1, but also with a reductant-activated pertussis holotoxin (Fig. 4*C* and *D*). The auto-ADP-ribosylated residues in wt rPtxS1 were mapped by mass spectrometry (Fig. 4*E*, S2). Three amino acids at the NAD+ binding site with nucleophilic sidechain functional groups (Cys41, SH-group; Thr125, OH-group; and Tyr126, OH-group) were found to be mono-ADP-ribosylated. Given the relatively long distance of these amino acids from the anomeric 1′ carbon of β-NAD+ (Fig. 4*E*), it appears that the nucleophilic amino acids either frequently reach a close distance to the electrophilic ribose 1′ carbon due to protein conformational fluctuation or there is significant movement of the highly reactive oxocarbenium cation intermediate at the NAD+ binding site of PtxS1. In any case, the auto-ADP-ribosylation of amino acids at the NAD+ binding site has potential to limit the catalytic activity of PtxS1 by sterically interfering with β-NAD+ binding.

### Detection of BaAD formation by the catalytic activity of PtxS1

**Figure 5:**
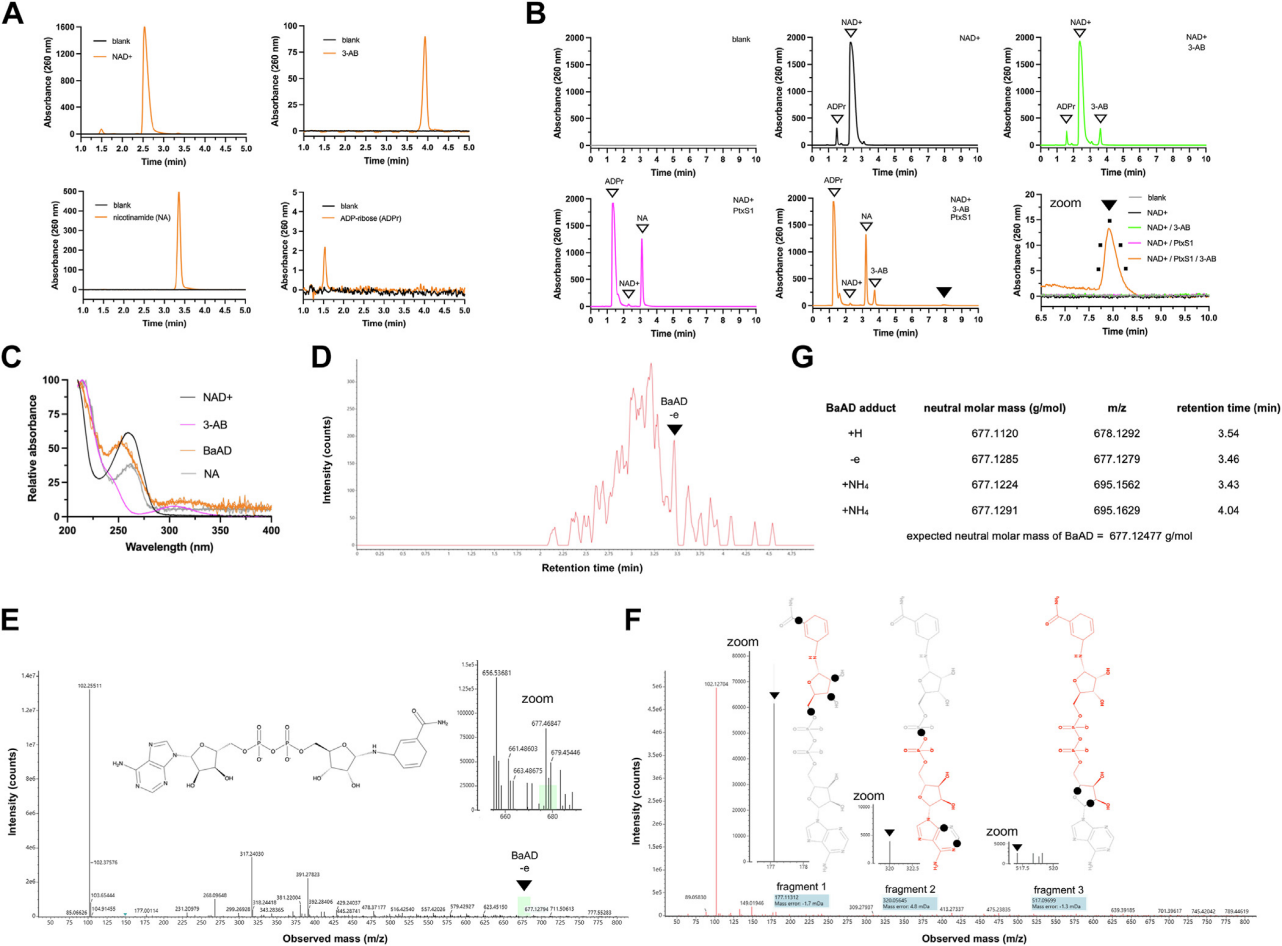
**Mass spectrometry-based analysis of PtxS1-catalyzed BaAD formation.***A*, ability of the HPLC method B to separate NAD+, 3-AB, nicotinamide (NA), and ADP-ribose. *B*, HPLC traces of SEC-purified wt rPtxS1 (200 μM) reaction after incubation with different reaction components for 72 h at room temperature, and filtration with a 10 kDa cutoff centricon. The *arrow marks* the position of a peak at around 8 min retention time that only appeared when wt rPtxS1 was incubated together with 3-AB (20 mM) and NAD+ (2 mM). The five dots in the subpanel “zoom” mark the positions where spectral scans were executed to analyze the homogeneity of the eluting compound. *C*, peak purity spectral scan analysis of the compound eluting at ca. 8 min and control nucleotides. The spectral scans were executed five times per peak. *D*, chromatogram of LC-MS analysis. The ca. 8 min peaks from multiple HPLC runs (see subpanel B) were pooled and analyzed with MS. *E*, mass spectrum with 30V cone voltage. This mass spectrum example is shown for the identification of the -e adduct of BaAD (see subpanel G). *F*, mass spectrum with fragmentation 60 to 120 V cone voltage. This mass spectrum example is shown for the identification of the -e adduct of BaAD (see subpanel G). Three example fragments are shown, which led into identification of BaAD from the ca. 8 min eluting peak (see subpanel B). *G*, different adducts of BaAD detected in the MS runs. BaAD, benzamide amino adenine dinucleotide; PtxS1, pertussis toxin S1 subunit; SEC, size-exclusion chromatography.

Next, we turned our attention to analyze whether we can detect the formation of BaAD by an orthogonal assay that would support our protein crystallization data (10). We incubated the C41S mutant of rPtxS1 in the presence or absence of different combinations of 3-AB and β-NAD+, and measured the reaction components with HPLC using a 260 nm UV detector (Figs. S3 and S4, HPLC method A). Prior to injection, the reactions were passed through a 10 kDa cutoff centricon filters, *i.e.*, we injected to HPLC nonprotein bound reaction components. We witnessed a distinct peak with ca. 7.25 retention time that was missing from the controls (Fig. S3*C*). As shown in Figure S3*D* and Figure S4, we witnessed the ca. 7.25 retention time peak also with the wt as well as with the C41G mutant of rPtxS1, but not with the NAD+ consumption-deficient Q127D/E129D double mutant of rPtxS1. Therefore, the presence of this ca. 7.25 retention time peak was dependent on the ability of rPtxS1 to hydrolyze β-NAD+. Also, when we analyzed the reaction components still present in the proteins trapped to the 10 kDa centricon filters, we did not detect the ca. 7.25 retention time peak (Fig. S3*E*). This indicates that the ca. 7.25 retention time peak compound is mostly getting released into solution after its formation. The formation of the ca. 7.25 retention time peak component also correlated with the rPtxS1 concentration, and the amount of 3-AB in the reaction mixture (Fig. S3*F*). Next, we focused on improving the separation of the reaction components in HPLC. As shown in Figure 5*A*, the C18 matrix with isocratic elution mobile phase (HPLC method B) resulted in clear separation of NAD+, nicotinamide, 3-AB and ADP-ribose (Fig. S3, *A and B* with HPLC method A in comparison). When NAD+ and 3-AB were incubated with rPtxS1, we witnessed a distinct peak with ca. 8 min retention time, far from the other reaction components, that was missing from the controls (Fig. 5*B*). Spectral scan analyses indicated that the ca. 8 min peak was composed of a single compound (Fig. 5*C*). To study the molecular nature of the ca. 8 min eluting compound, we pooled material of different HPLC injections and ran an LC-MS analysis (Fig. 5*D*–*G*). The LC-MS analysis led into identification of BaAD with three different adducts (+H, -e, and +NH_4_) in multiple independent runs. Next, having established the identity of the ca. 8 min eluting compound as BaAD, we estimated the biocatalytic yield. Based on an area under curve (AUC) analysis, we estimated that the amount of BaAD was ca. 0.5%, as in proportion to the original NAD+ inoculum. Using UV absorbance of β-NAD+ solution as a reference at two different wavelengths, we estimated that one representative BaAD preparate (30 μl) contained 631 μM (254 nm absorbance) or 627 μM (260 nm absorbance) of BaAD. Taken together, the HPLC and LC-MS data demonstrate that rPtxS1 catalytically produces BaAD and that the formed BaAD is mostly getting released into solution allowing its subsequent purification.

### Modeling of BaAD analog formation by PtxS1

**Figure 6:**
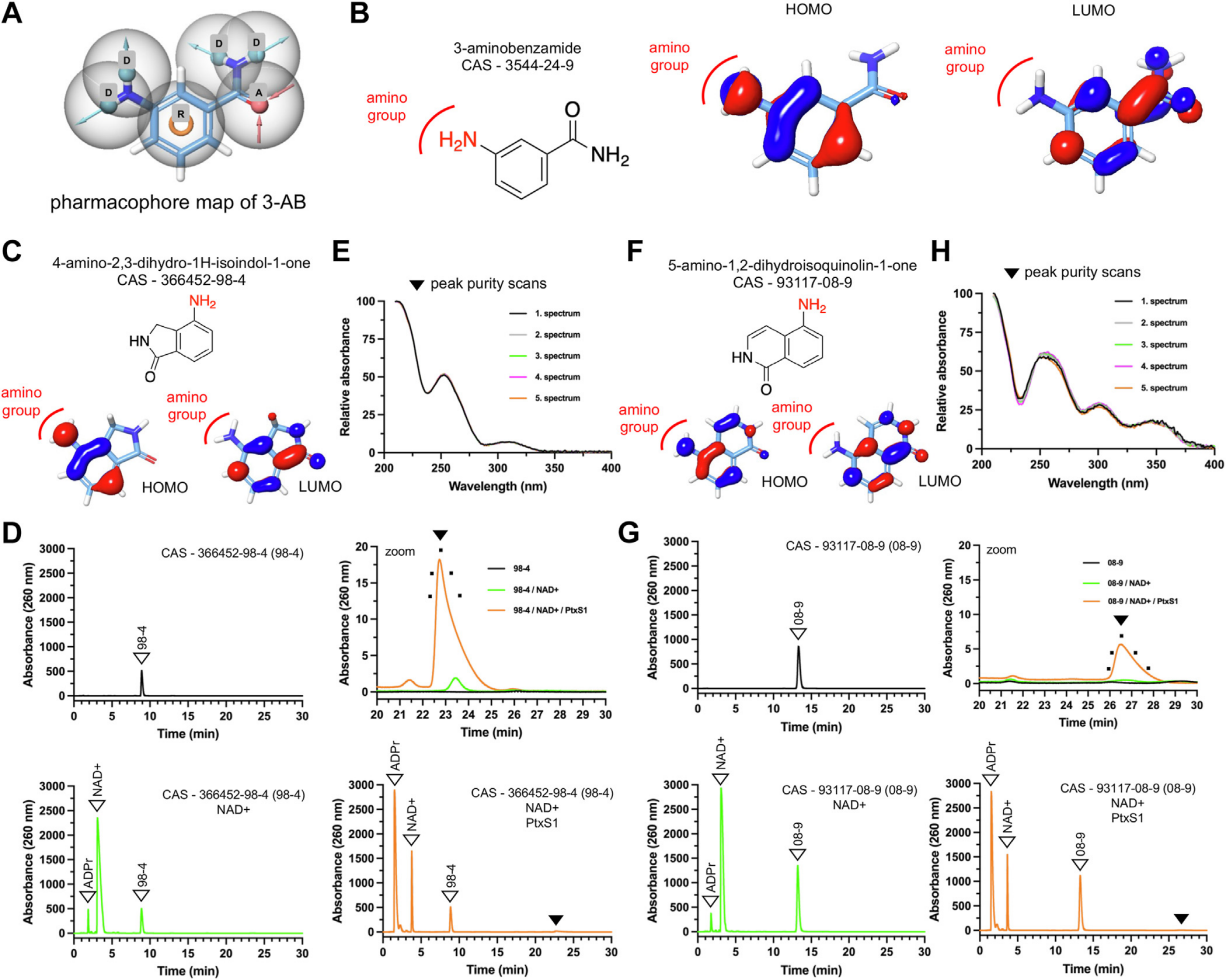
**Detection of BaAD analog formation by PtxS1.***A*, pharmacophore map of 3-AB showing four hydrogen bond donor (D) characters on amino group and amide nitrogen respectively, two hydrogen bond acceptors (A) on carbonyl group and 1-ring (R) character on central phenyl ring. *B*, the 2-dimensional (2D) structure of 3-AB and its representation in 3-dimension (3D) after geometry optimization by quantum mechanical (QM) simulation. The *red* and *blue* surfaces show the HOMO and LUMO energies calculated from QM simulations. *C*, the 2-D structure of 4-amino-2,3-dihdro-1H-isoindol-1-one (compound 98–4) analog of 3-AB and its geometry optimized structure depicting HOMO and LUMO energies after QM simulations. *D*, HPLC chromatogram of compound 98–4 at its RT ∼9 in *black*. HPLC chromatogram of compound 98–4 along with β-NAD+ and its degradation product ADPr in absence of rPtxS1 protein in *green* after 72 h incubation at RT. The HPLC chromatogram of compound 98–4 along with β-NAD+ and its degradation product ADPr in presence of rPtxS1 protein in orange after 72 h incubation showing the formation of BaAD like analog. Zoom-in view of HPLC chromatogram confirming the formation of BaAD like compound at RT ∼23 which is complete absent in plain chromatogram of 98–4 and forms in very small amount in absence of recombinant PtxS1 when incubated with β-NAD+. *E*, peak purity profile of BaAD like analog obtained from compound 98–4 after incubation with β-NAD+ and rPtxS1. *F*, the 2D structure of 5-amino-1,2-dihdroisoquinoline-1-one (compound 08–9) analog of 3-AB and its geometry optimized structure depicting HOMO and LUMO energies after QM simulations. *G*, HPLC chromatogram of compound 08 to 9 at its RT ∼14 in *black*. HPLC chromatogram of compound 08 to 9 along with β-NAD+ and its degradation product ADPr in absence of rPtxS1 in *green* after 72 h incubation at RT. The HPLC chromatogram of compound 08 to 9 along with β-NAD+ and its degradation product ADPr in presence of rPtxS1 in *orange* after 72 h incubation showing the formation of BaAD like analog. Zoom-in view of HPLC chromatogram confirming the formation of BaAD like compound at RT ∼26 which is complete absent in plain chromatogram of 08 to 9 and forms in negligible amount in absence of recombinant PtxS1 and incubated with β-NAD+. *H*, peak purity profile of second BaAD like analog obtained from compound 08 to 9 incubated with β-NAD+ and rPtxS1. BaAD, benzamide amino adenine dinucleotide; PtxS1, pertussis toxin S1 subunit; RT, room temperature.

We hypothesized that PtxS1 could provide a more universal biocatalytic platform to produce nonhydrolyzable NAD+ analogs. Therefore, we executed a pharmacophore-based screening on 3-AB followed by quantum mechanical (QM) simulations. We attempted to identify analogs of 3-AB, which would have the capacity to insert into the nicotinamide pocket of PtxS1 (see Fig. 1*B*), and also would have the amino group-associated nucleophilic power to attack the PtxS1-formed oxocarbenium cation. We identified four unique pharmacophore features of 3-AB and built the model (Fig. 6*A*), *i.e.*, two hydrogen bond donors on each amine and amide nitrogen, two hydrogen bond acceptors on amide oxygen, and one aromatic ring. The hypothesis was validated by using two known PARP inhibitors Veliparib and Iniparib as actives against 16,428 decoys from dude diverse dataset as inactives (20). The Boltzmann-enhanced discrimination receiver operator characteristic (ROC) AUC value was 0.661 (alpha=20.0, alpha∗Ra=0.0024), which is bounded between 1 and 0, with 1 being ideal screen performance. The ROC AUC value were also measured, which is bounded between 1 and 0, with one being ideal screen performance and 0.5 reflecting random behavior. Our hypothesis shows ROC of 0.97 reflecting robust model, which was further confirmed by large robust initial enhancement value of 13.20 indicating better screen performances. Finally, the hypothesis was used to screen the zinc database compound library. After screening, the compounds were ranked, and structural analysis was performed. We hypothesized that bulky side chains may not be appropriate to bind into small nicotinamide binding pocket on PtxS1 (Fig. 1*B*) hence these structures were not considered for further evaluation. The final outcome was identification of 277 potential compounds, which were then submitted for QM simulations. The 277 selected molecules were subjected to geometric optimization to generate minimum energy conformations. These low energy conformations obtained were then predicted for HOMO and LUMO energies, followed with the gap energy value calculations. Table S1 and Figure S5 describe the 14 analogs of 3-AB that were selected for experimental analysis based on the pharmacophore-based screening and QM simulations.

### Experimental detection of BaAD analog formation by PtxS1

First, we analyzed if the 14 analogs of 3-AB interfere with the ART activity of PtxS1 by measuring PtxS1-catalyzed ADP-ribosylation of its Gαi substrate protein. As shown in Figure S6, none of the compounds had major inhibitory effects even when assayed at 200 μM concentration. Therefore, we next did coincubations with all the 3-AB analogs, wt rPtxS1, NAD+ and measured the reaction components with HPLC using a 260 nm UV detector. Prior to injection, the reactions were passed through a 10 kDa cutoff centricon filters, *i.e.*, we injected to HPLC nonprotein bound reaction components. We witnessed distinct peaks with two 3-AB analogs out of the 14 tested compounds, far from the initial reaction components, that were missing from the controls (Fig. 6*D* and *G*). Spectral scan analyses indicated that these two peaks were composed of single compounds (Fig. 6*E* and *H*). To study the molecular nature of these two compounds, we pooled material of different HPLC injections and ran an LC-MS analysis (Fig. S7). The masses of the detected compounds closely matched the theoretical sizes of compounds that could have been formed from oxocarbenium cation and 4-amino-2,3-dihydro-1H-isoindol-1-one or 5-amino-1,2-dihydroisoquinolin-1-one at the NAD+ binding site of PtxS1 (Fig. S7). We name these BaAD analogs here as isoindolone amine adenine dinucleotide (IiaAD), and isoquinolinone amine adenine dinucleotide (IqaAD). To further characterize the new compounds, we studied their fluorescence properties together with BaAD. The IqaAD, but not BaAD or IiaAD, showed significant fluorescence emission with a maximum emission at 414 nm when excited with 330 nm (Fig. S8). This paralleled the fluorescence properties of the 5-amino-1,2-dihydroisoquinolin-1-one precursor of IaqAD. Having established the identity of IiaAD and IqaAD, we estimated their biocatalytic yield. Using UV absorbance of β-NAD+ solution as a reference at two different wavelengths, we estimated that one representative IiaAD preparate (30 μl) contained 853 μM (254 nm absorbance) or 823 μM (260 nm absorbance) of IiaAD. One representative IqaA preparate (30 μl) contained 297 μM (254 nm absorbance) or 293 μM (260 nm absorbance) of IqaAD. Overall, the HPLC and LC-MS data demonstrate that rPtxS1 catalytically produces two different analogs of BaAD and that the formed BaAD analogs are getting released into solution allowing their subsequent purification.

### Neglectable hydrolysis of BaAD and analogs

We adopted a framework based on the International Council for Harmonisation of Technical Requirements for Pharmaceuticals for Human Use (ICH) guidelines [ICH Q1A(R2)] for forced degradation studies of new drug substances and products to evaluate the hydrolytic stability of BaAD, IiaAD, and IqaAD. First, to establish initial conditions for acid- or base-catalyzed hydrolysis, β-NAD+ was incubated in 0.1 M HCl, 0.1 M NaOH, and MQ-H_2_O, and analyzed with HPLC after 2 h, 24 h, and 72 h. The highest degree of β-NAD+ hydrolysis to ADP-ribose and nicotinamide was observed in samples incubated with 0.1 M NaOH for 72 h (Fig. S9). Next, the 72 h BaAD, IiaAD, and IqaAD biocatalysis reactions were incubated in 0.1 M NaOH for 72 h, and analyzed with HPLC. The amount of BaAD, IiaAD, and IqaAD did not decrease upon incubation in high pH (Figs. S10 and S11). In fact, the AUC analysis of BaAD and IiaAD peaks indicated that more of these compounds were present after incubation in 0.1 M NaOH for 72 h. Moreover, HPLC analysis of the high pH treated rPtxS1-free BaAD and IiaAD biocatalysis reaction controls led into detection of BaAD and IiaAD (Figs. S10 and S11). This observation implies that hydrolysis of β-NAD in 0.1 M NaOH generates a chemical entity, most likely an oxocarbenium cation similar to PtxS1 catalysis, which reacts with the precursors of BaAD and IiaAD. Overall, the hydrolysis studies in high pH indicate that BaAD, IiaAD, and IqaAD are nonhydrolyzable.

## Discussion

Enzyme promiscuity is the ability of an enzyme to catalyze an unexpected side reaction in addition to its main reaction. Recently, we detected binding of a novel chemical entity that resembles NAD+ at the NAD+ binding site of pertussis toxin PtxS1 subunit (10). We named this chemical entity as BaAD. The binding pose was acquired after crystallization of a C41S-mutant of the truncated form of PtxS1 (C41S-rPtxS1) with NAD+ and 3-AB. Here, we set out to confirm the formation of BaAD by PtxS1, to investigate the structural and mechanistic basis of its formation, and to assess the feasibility of PtxS1-mediated biocatalytic process to produce BaAD for downstream applications.

Compelling evidence was obtained on PtxS1-catalyzed formation of BaAD. We witnessed a distinct peak in two different HPLC methods that was missing from the controls of rPtxS1 incubations with NAD+ and 3-AB. The ca. 7.25 min HPLC method A peak was evident with wt-rPtxS1, C41S-rPtxS1 and C41G-rPtxS1, but not with Q127D/E129D-rPtxS1, which has a radically reduced NADase and ART. The height of the ca. 7.25 min HPLC peak also correlated with the amounts of PtxS1 and 3-AB in the reaction. Furthermore, the ca. 7.25 min peak was witnessed in the 10 kDa cutoff filtrates of the wt-, C41S-, and C41G-rPtxS1 reactions, not in the proteins trapped to the centricon filters. The HPLC method B with isocratic elution mobile phase turned out to be superior due to its ability to clearly resolve all the reaction components. When the material in the ca. 8 min HPLC method B peak was analyzed with mass spectrometry, three different adducts (+H, -e, and +NH_4_) of BaAD were detected in multiple independent runs. The neutral molar masses ranged from 677.1120 to 677.1291 g/mol, which closely matched the expected neutral molar mass of BaAD (677.12477 g/mol). The detection of numerous BaAD matching fragments with the high cone voltage energy supported the notion that the molecular identity of the ca. 8 min eluting compound was indeed BaAD. We conclude that rPtxS1 catalytically produces BaAD, and that the formed BaAD is mostly released into solution. However, BaAD has clear affinity to the NAD+ binding site of rPtxS1 supported by our current modeling exercise as well as our 1.0 Å resolution PtxS1/BaAD cocomplex structure that we recently published (10). It is plausible that the hampered NADase activity of C41S-rPtxS1 with spatial NAD+ binding site alterations contributed to the success with C41S-rPtxS1/BaAD cocomplex crystallization.

**Figure 7:**
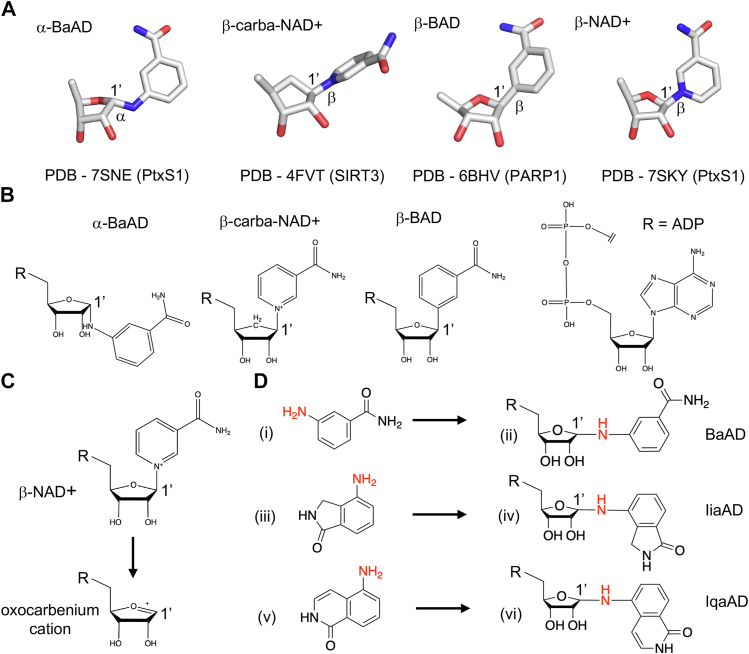
**Formation mechanism of BaAD in PtxS1.***A*, comparison of BaAD structure with two known nonhydrolyzable NAD+ analogs. Experimentally determined binding poses of the compounds in different proteins are displayed. The stereochemistry of the bond from the 1′ anomeric carbon is highlighted for each compound. Oxygen and nitrogen atoms in *red* and *blue*, respectively. *B*, comparison of BaAD structure with two known nonhydrolyzable NAD+ analogs carba-NAD+ and BAD. Chemical structures of the compounds. The stereochemistry of the bond from the 1′ anomeric carbon is highlighted for each compound. *C*, possible stereospecific mechanism of BaAD formation in the NAD+ binding site of PtxS1. BAD, benzamide adenine dinucleotide; BaAD, benzamide amino adenine dinucleotide; PtxS1, pertussis toxin S1 subunit. *D*, chemical structures of BaAD, IiaAD (isoindolone amine adenine dinucleotide), IqaAD (isoquinolinone amine adenine dinucleotide), and their precursors.

In our 1.0-Å resolution BaAD/PtxS1 structure (10), the glycosidic bond between the anomeric 1′ carbon, and the nitrogen of 3-AB is in alpha configuration (Fig. 7*A* and *B*). The alpha configuration is different as compared to the two commercially available nonhydrolyzable NAD+ analogs, carba-NAD+, and BAD, which both have the same beta configuration stereochemistry as with the natural substrate NAD+ (Fig. 7*A* and *B*). It is plausible that the formation of α-BaAD by PtxS1 involves the canonical stereospecific ART-catalyzed reaction producing α-ADP-ribosyl linkages from β-NAD+ (Fig. 7*C*), as witnessed already in a number of early ART studies, *e.g.*, (21, 22, 23). Accordingly, we envision that PtxS1/β-NAD+ -complex first undergoes activation of β-NAD+ to form the reactive oxocarbenium cation (24). Next, nucleophilic attack of the electrophilic ribose 1′ carbon of the oxocarbenium cation takes place by the nitrogen atom of the 3-AB amino group, instead of the oxygen atom of water molecule or the sulfur atom of a cysteine side chain of the human Gαi, which is the natural substrate protein for PtxS1 (25, 26, 27). The nucleophilic attack could proceed *via* nicotinamide pocket-bound 3-AB, *i.e.*, nicotinamide has been replaced by 3-AB after the oxocarbenium cation formation. Alternatively, the nucleophilic attack could occur by free solution form of 3-AB followed by BaAD 3-AB remnant insertion into the nicotinamide pocket, which would at that very moment either be nicotinamide-free or become nicotinamide-free due to BaAD 3-AB remnant insertion. It is possible, however, that BaAD could be formed after the oxocarbenium cation has moved out of its initial formation binding pose, either with a nicotinamide pocket-bound or free solution form of 3-AB. Based on our modeling exercise, oxocarbenium cation has a lower affinity as compared to NAD+ at the NAD+ binding site and thereby potential to move from its initial formation binding pose. Also, we experimentally detected three nucleophilic amino acids (Cys41, Tyr126, and Thr125) of the rPtxS1 NAD+ binding site to be auto-ADP-ribosylated that are relatively far from the position of the electrophilic ribose 1′ carbon (Fig. 4*E*) (10). The closest atomic distance is 8.2 Å between the sulfur atom of Cys41 and the electrophilic ribose 1′ carbon. Accordingly, PtxS1 might in fact create a diffusible cloud of oxocarbenium cations, which would react with any suitable nucleophile even across long distances, *e.g.*, water molecule, nucleophilic amino acids such as Cys41 or the amino group of 3-AB. However, we regard this mechanistic scenario in BaAD formation somewhat questionable as the lifetime of oxocarbenium cation is short as in picoseconds (24). Also, there is currently no evidence showing that the NAD+ binding site of PtxS1 would be able to exclude water, which is one potent oxocarbenium cation attacking nucleophile quickly yielding chemically inert ADP-ribose from the oxocarbenium cation. Furthermore, we cannot be sure that the detected automodified residues in PtxS1 are result of a catalytic process within single PtxS1 molecules (cis) and not of a catalytic process between different PtxS1 molecules (trans), or even between PtxS1 and some unknown endogeneous *E. coli* ART during overnight expression prior to purification and biochemical experimentation. Overall, we propose here that rPtxS1 catalytically produce α-BaAD from β-NAD+ and 3-AB involving a process where the oxocarbenium cation intermediate stays relatively static at its initial position of formation thereby allowing efficient stereospecific attack of the electrophilic ribose 1′ carbon by 3-AB.

We hypothesized that PtxS1 could provide a more universal biocatalytic platform to produce nonhydrolyzable NAD+ analogs. Therefore, we executed a pharmacophore-based screening on 3-AB followed by QM simulations, which offer the possibility for a detailed description of the interactions between electrons and orbitals in the breaking and forming of new bonds, *e.g.*, in reactions between the PtxS1-formed oxocarbenium cation and 3-AB. The modeling approach based on a 3-AB pharmacophore led us to experimentally validate 14 commercially available analogs of 3-AB with HPLC and mass spectrometry experiments. We witnessed distinct peaks with two 3-AB analogs, far from the initial reaction components, that were missing from the controls. The masses of the detected compounds closely matched the theoretical sizes of compounds that could have been formed from oxocarbenium cation and 4-amino-2,3-dihydro-1H-isoindol-1-one, or 5-amino-1,2-dihydroisoquinolin-1-one at the NAD+ binding site of PtxS1. We name these BaAD analogs here as IiaAD, and IqaAD. It is noteworthy that IqaAD was strongly fluorescent, which paralleled the fluorescence properties of the parental 3-AB analog. This property potentially widens the applicability of IqaAD to include, for example, competitive fluorescence polarization-based assays to screen small molecular weight compound inhibitors of ARTs.

Identification of efficient biocatalytic processes to produce nonhydrolyzable NAD+ analogs would be highly advantageous. The nonhydrolyzable NAD+ analogs carba-NAD+ and BAD, which are now commercially available, are expensive to purchase in large quantities, and not widely used, despite their clear benefits in structural elucidation of the NAD+ binding modes to proteins. Biocatalytic processes also would limit the use of industrial chemical processes that would be in line with the global sustainable development goals. However, based on our HPLC chromatograms (Figs. 5 and 6), the yields of our typical BaAD, IiaAD, and IqaAD were relatively low in respect of the precursor amounts. Our most optimal reaction setups used rPtxS1 concentration (200 μM) at the upper limit of rPtxS1 solubility, as well as 3-AB/3-AB analog concentration (20 mM) at the near possible maximum, which already starts to inhibit the enzyme (10). Also, during the catalytic process, nicotinamide and ADP-ribose are getting formed, which occupy the NAD+ binding site, and thereby most likely interfere with BaAD, IiaAD, and IqaAD formation. We therefore think that in-solution PtxS1-mediated biocatalysis of α-BaAD cannot be significantly optimized. Acquisition of sufficient amount of material for downstream applications simply needs replicates, which is supported by the fact that purification of rPtxS1 is easy, and all the precursor molecules are cheap and easily accessible. In addition, covalent or noncovalent immobilization of rPtxS1 to a solid carrier particle or a surface (28) could allow more control with cyclical spiking of reaction components and product retrieval thereby possibly resulting in more efficient production yields. The only major catalysis-limiting factor in solid-phase could be the auto-ADP-ribosylation of amino acids at the NAD+ binding site of PtxS1 that was detected in this study. It appears likely that the ADP-ribose attached to the amino acids of the NAD+ binding site would limit the catalytic activity of PtxS1 by sterically interfering with β-NAD+ binding. Other ARTs and NAD+ consuming enzymes, *e.g.*, the efficient NADase tuberculosis necrotizing toxin (3), or the *E. coli* pertussis-like toxin (29), could provide alternative enzymatic basis for BaAD and BaAD analog production. In the end, these enzymes rely in their catalytic process on the production of oxocarbenium cation intermediate. We envision that whenever there is an enzyme that is capable of forming the oxocarbenium cation from β-NAD+ in sufficient quantities, and additionally to accommodate 3-AB/3-AB analog into the NAD+ binding site close to the electrophilic ribose 1′ carbon, there is a possibility that nonhydrolyzable NAD+ analogs are formed. Overall, we have confirmed the previous indications (10) that the PtxS1 ART subunit of pertussis toxin catalytically produces a new kind of an NAD+ analog BaAD from β-NAD+ and 3-AB. We also extended the concept of PtxS1-based biocatalysis to produce other nonhydrolyzable NAD+ analogs. The new biocatalytically produced NAD+ analogs emerge as valuable tools to study the structural biology and enzymology of NAD+ binding and NAD+ consuming enzymes, such as human PARPs and bacterial ART exotoxins. As the first proof of concept of broader applicability, we detected that IiaAD, but not BaAD, inhibited the NAD+ consumption activity of human PARP10 (Fig. S12). This finding was supported by our molecular modeling exercise (Fig. S13). The new nonhydrolyzable NAD+ analogs pave the way to speed up drug development efforts targeting the NAD+ binding sites of eukaryotic and prokaryotic ARTs *via* experimental molecular visualization techniques such as X-ray crystallography.

## Experimental procedures

### Molecular modeling

(i) Method: The Schrödinger drug discovery suite (Release 2023–1: Schrödinger, LLC, New York, NY, 2023) was used for computational modeling, including structure preparation, ligand docking, binding free energy predictions, MD simulations, and visualizations. Pymol version 2.4.1 (https://pymol.org) and ChimeraX 1.3 (https://www.cgl.ucsf.edu/chimerax/download.htlm) were also used for supplementary visual representation of the results. (ii) Molecular docking: Molecular docking was performed using the extra precision (XP) mode of Glide software (https://www.schrodinger.com/platform/products/glide/) (30) (Schrödinger Release 2023–1: Glide, Schrödinger, LLC, New York, NY, 2023). The docking was carried out at the β-NAD+ binding site of PtxS1, which was defined by the cocrystallized β-NAD+ and α-BaAD structures (10). For the apo structure of PtxS1 (19), the autoinhibitory C-terminal region, which occupies the NAD+ binding site and is stabilized with a Cys41-Cys202 disulfide bond, was removed starting from Val222 (Uniprot P04977). The *in silico* truncated PtxS1 apo protein structure was aligned with the cocrystallized β-NAD+ structure (10), and a docking grid box was generated around the β-NAD+ binding site. For PARP10 the docking was carried out using cocrystallized structure of PARP10 and 3-AB (PDB 6FXI, Uniprot Q53Gl7). The outer box dimensions were set to 30 Å × 30 Å × 30 Å. Flexible ligand sampling was used, and for each ligand, 10 poses were generated for postdocking minimization. Finally, one pose per ligand was selected based on the highest docking score, which included the addition of Epik state penalties (31). (iii) Binding free energy prediction: The Prime/MM-generalized born surface area (GBSA) tool of Maestro software (https://www.schrodinger.com/platform/products/maestro) (Schrödinger Release 2023–1: Prime, Schrödinger, LLC, New York, NY, 2023) was used to calculate the binding free energies for the docked poses of the compounds. The binding free energy (ΔG_bind_) was estimated by Prime/MM-GBSA method (32, 33) using the equation ΔG_bind_ = ΔE_MM_ **+** ΔG_solv_ **+** ΔG_SA_ where ΔE_MM_ represents the difference in energy between the complex structure and the sum of the energies of the ligand and unliganded receptor (using the OPLS4 molecular mechanics force field (34)), ΔG_solv_ represents the difference in the GBSA solvation energy of the complex and the sum of the solvation energies for the ligand and unliganded receptor (calculated with the VSGB2.1 solvation model (35)), and ΔG_SA_ represents the difference in the surface area energy for the complex and the sum of the surface area energies for the ligand and uncomplexed receptor.(iv) MD simulation: MD simulations were conducted using the Desmond software (https://www.schrodinger.com/platform/products/desmond) (Schrödinger Release 2023–1: Desmond Molecular Dynamics System, D. E. Shaw Research, New York, NY, 2023. Maestro-Desmond Interoperability Tools, Schrödinger, New York, NY, 2023) with the OPLS4 force field. The purpose of the simulations was to study the behavior of the ligand-PtxS1/PARP10 complexes over a time period of 500 ns. The simulation systems were prepared using the System Builder tool of Desmond. An orthorhombic simulation box with periodic boundary conditions was generated to create an environment resembling bulk solution. The solute, which consisted of the ligand-enzyme complex, was solvated with single point charge water molecules. To prevent artificial interactions between the solute and its periodic images, a 10 Å buffer space was maintained between the solute and the box edges. Additionally, sodium and chloride ions were added to the system to achieve neutralization and a concentration of 15 mM. The simulation system underwent relaxation to equilibrate the solute and the surrounding water molecules. Two relaxation steps were performed - one with restraints on the solute heavy atoms and another without restraints. In the first step, a stepwise relaxation protocol was used. The system was initially subjected to 12 ps of Brownian dynamics in the NVT ensemble at 10K, using a Berendsen thermostat and a 1-fs time step, while applying restraints to the solute. This was followed by 12 ps of Langevin dynamics in the NPT ensemble at 300 K and 1 atm pressure, using a Berendsen thermostat and barostat, with restraints applied to the solute. In the final relaxation step, the system was allowed to relax for 24 ps without any restraints. After relaxation, the production simulations were carried out in the NPT ensemble for a duration of 500 ns, using a 2-fs time step. The temperature was maintained at 300 K, using the Nosé-Hoover chain thermostat (36, 37), while the pressure was maintained at 1.01325 bar using the Martyna-Tobias-Klein barostat (38). The pressure and temperature relaxation times were set to 1 ps and 2 ps, respectively, with isotropic coupling. To handle short-range Coulombic interactions, a cutoff radius of 9.0 Å was used. The long-range electrostatic interactions were calculated using the u-series decomposition of the Coulomb potential (39). The resulting simulation trajectories were analyzed using the Simulation Interactions Diagram tool in Maestro. The data generated from the interaction analysis were further processed using Microsoft Excel 365 (https://www.office.com) to create graphical representations for detailed analysis and interpretation.

### Pharmacophore-based modeling

(i) Ligand preparation and phamacophore modeling: The pharmacophore modeling was performed using Phase (Schrödinger Release 2022–4: Jaguar, Schrödinger, LLC, New York,NY, 2022). The 3D structure of 3-AB was used to generate the pharmacophore model. The compound was imported along with other PARP inhibitors from pubchem database into Maestro workspace (Schrödinger Release 2022–4: Maestro, Schrödinger, LLC, New York, NY, 2022). Using LigPrep tool the structures were desalted and possible tautomeric states were generated at pH 7.0  ± 2.0 with Epik. The OPLS4 force field was applied to generate optimized low-energy 3D conformers. The following pharmacophore features used included the acceptor (A), donor (D), and aromatic ring (R) generating four-point pharmacophore model hypotheses. Various statistical parameters such as the enrichment factor, Boltzmann-enhanced discrimination receiver operator characteristic area under the curve (40), Receiver Operator Characteristic AUC, robust initial enhancement, and area under the accumulation curve were used to measure the performance of the hypothesis by distinguishing between active and inactive compounds. After validation, the final four-point model used for pharmacophore screening of compound library taken from Zinc database. (ii) QM simulations and pharmacokinetic properties predictions. The 3D structures of the screened compounds along with 3-AB were initially optimized in the OPLS4 force field and were subjected to *ab initio* QM geometry optimization using Jaguar (Schrödinger Release 2022–4: Jaguar, Schrödinger, LLC, New York,NY, 2022 (41)). The 6–31G∗∗ basis set was selected with the B3LYP-D3 density functional theory (42). The automatic self-consistent field spin treatment using medium grid density with nonrelativistic Hamiltonian was performed. Geometry optimization in the gas phase was carried out for 100 steps followed by further optimization in Poisson Boltzmann Finite solvation model in water with default convergence criteria. The highest and lowest occupied molecular orbitals (HOMO and LUMO, respectively) were calculated. The Schrödinger’s QikProp tool (Schrödinger Release 2022–4: QikProp, Schrödinger, LLC, New York, NY, 2022) was used to predict pharmacokinetic properties of the selected compounds.

### Protein expression and purification

Plasmid constructs for *E. coli* expression of recombinant PtxS1 (wt and Q127D/E129D mutant) and human Gαi have been described (2). Synthetic DNA fragments (Eurofins Genomics) encoding for Cys41Gly and Cys41Ser mutants of PtxS1 were cloned into pET15b (Novagen) with NdeI and BamHI as previously with wt PtxS1 (2). Expression and purification of the PtxS1 and Gαi proteins with immobilized metal affinity chromatography and size-exclusion chromatography (SEC) was conducted as described (2). However, the final buffer composition of the SEC-purified proteins differs, *i.e.*, we now used 100 mM Hepes (pH 7.5), 500 mM NaCl, 10% (w/v) glycerol buffer with additional 2 mM DTT in the case of rPtxS1. Whenever immobilized metal affinity chromatography-purified rPtxS1 proteins were used in the experiments, the imidazole-containing buffer was exchanged to 100 mM Hepes (pH 7.5), 500 mM NaCl, 10% (w/v) glycerol, and 2 mM DTT buffer using 10 kDa cutoff centricon filter units (Merck Milipore). A synthetic DNA fragment (Eurofins Genomics) encoding for the catalytic domain of human PARP10 (N819-V1009–PARP10-Cat, Uniprot Q53Gl7) was cloned with ligation independent cloning strategy into pNIC28-Bsa4 (Structural Genomics Consortium). The PARP10-Cat expression plasmid was transformed into BL21(DE3) (Novagen) and selected overnight at 37 °C on LB agar with 50 μg/ml kanamycin. Next morning, the bacterial lawn from the LB-plates was transferred into Terrific broth autoinduction medium (Formedium, AIMTB0205) supplemented with 0.8% (w/v) of glycerol with 50 μg/ml kanamycin. Cultures were grown at 37 °C with 250 rpm until absorbance at 600 nm reached 1 (typically 3–5 h), and temperature was reduced to 18 °C. Bacteria were collected after 24 h by centrifugation and were frozen to −80 °C as pellets. The bacteria were thawed on ice in 100 mM Hepes (pH 7.5), 500 mM NaCl, 10% (w/v) glycerol, 2 mM DTT buffer containing 10 mM imidazole, EDTA-free Protease Inhibitor Tablets (Thermo Fisher Scientific, A32965, 1 tablet/50 ml) and 0.5 mg/ml lysozyme (Sigma-Aldrich, L6876). Samples were sonicated and clarified by centrifugation. The supernatant was mixed with 5 ml of Protino Ni-NTA Agarose (Macherey-Nagel, 745400) settled with the lysis buffer. The mixture was incubated for 1 h at 4 °C in an end-over-end shaker. The resin was washed batch-wise with centrifugation and in a plastic column with gravity flow using 20 mM imidazole in 100 mM Hepes (pH 7.5), 500 mM NaCl, 10% (w/v) glycerol, and 2 mM DTT buffer. PARP10-Cat was eluted with 500 mM imidazole in 100 mM Hepes (pH 7.5), 500 mM NaCl, 10% (w/v) glycerol, 2 mM DTT buffer, and subjected to SEC on HiPrep 16/60 Sephacryl S-100 HR column (GE HealthCare) using 25 mM Hepes (pH 7.5), 300 mM NaCl, 10% (w/v) glycerol, and 0.5 mM tris(2-carboxyethyl)phosphine hydrochloride buffer. All the purified proteins were stored in −80 °C.

### Differential scanning fluorimetry

Folding properties of wt and mutant rPtxS1 were analyzed based on their thermal stability in a DSF experiment. The proteins at a concentration of 0.2 mg/ml were analyzed in 20 mM Hepes (pH 7.5), 500 mM NaCl, 10% (w/v) glycerol, 2 mM DTT. Samples were incubated with 5 × SYPRO Orange (Thermo Fisher Scientific, S6650) for 10 min, followed by a heating from 20 to 90 °C with 0.5 °C increments (1 min/1 °C) using a CFX96 Real-Time PCR detection system (Bio-Rad). Tm-values were determined using GraphPad Prism version 9.5.1 for Mac, GraphPad Software, Boston, Massachusetts USA, www.graphpad.com.

### NAD^+^ consumption assay

The reactions were performed in triplicates, either using flat-bottom 96-well black plates (Greiner Bio-One, Polypropylene 96-well F-bottom Microplates, Thermo Fisher Scientific, 10706172) or flat-bottom 384-well black plates (low binding surface-coated OptiPlate-384, Revvity, 6057261). Each reaction consisted of 1 to 10 μM of wt or mutant rPtxS1 with or without 10 μM NAD+ (Sigma-Aldrich, N3014) or 4 μM of PARP10-Cat with or without 10 μM NAD+ in 100 mM Hepes (pH 7.5), 250 mM NaCl, and 10% (w/v) glycerol. Whenever the inhibitory effect of different compounds was analyzed, the compounds (100 μM) were added 30 min before initiating the reaction with NAD+ addition. The reactions were carried out at room temperature with shaking at 300 rpm for 60 to 120 min, in a total reaction volume of 50 μl (96-well plate) or 20 μl (384-well plate). The reactions were stopped by adding 20 μl (96-well plate) or 8 μl (384-well plate) of 20% acetophenone (Sigma-Aldrich, A10701, diluted with ethanol) and 20 μl (96-well plate) or 8 μl (384-well plate) of 2 M KOH followed by incubation at room temperature for 10 min. Next, 90 μl (96-well plate) or 36 μl (384-well plate) of formic acid (Thermo Fisher Scientific, A117–500 or Merck, 5438040250) was added, and the plate was further incubated for 20 min wrapped in aluminum foil. The fluorescence readings were recorded with the VICTOR Nivo multimode plate reader (PerkinElmer), with excitation and emission wavelengths set at 355 nm ( ± 20 nm) and 450 nm ( ± 5 nm), respectively.

### ADP-ribosylation assays

(i) Recombinant PtxS1 experiments: Reactions (in 50 μl) contained 0.2 to 10 μM rPtxS1 wt or mutants, 2 to 10 μM biotinylated NAD+ (Trevigen, 4670–500–01) with or without 1 μM rGαi substrate in 100 mM Hepes (pH 7.5), 500 mM NaCl, 10% (w/v) glycerol, and 2 mM DTT. Whenever the inhibitory effect of different compounds was analyzed, the compounds were added after rPtxS1 and rGαi prior to initiating the reaction with biotin-NAD+ addition. The ADP-ribosylation reactions were carried out at room temperature for 40 min (canonical reaction) with shaking at 300 rpm or at 4 °C for 24 h (long reaction). Reactions were stopped by addition of Laemmli loading dye to 1x and heating for 10 min at 95 °C. The samples were run on SDS-PAGE and transferred to nitrocellulose membranes, followed by blocking with 1% (w/v) casein blocking buffer (Bio-Rad, 161–0782). Membranes were incubated with streptavidin conjugated to horse radish peroxidase (1:5000) (GE HealthCare, RPN1231VS) in 1% (w/v) casein blocking buffer (Bio-Rad, 161–0782) and washed thrice with Tris-buffered saline [10 mM Tris–HCl (pH 7.5) and 150 mM NaCl] containing 0.05% Tween 20 (TBST). Alternatively, after blocking with 4% (w/v) bovine serum albumin (BSA) in TBST, membranes were probed in TBST containing 2% (w/v) BSA for HIS-tagged rPtxS1 and rGαi with mouse monoclonal anti-HIS (1:1000) (R&D Systems, MAB050) or for ADP-ribose conjugates with a rabbit monoclonal Poly/Mono-ADP Ribose (E6F6A) (1:1000) antibody (Cell Signaling technology, 83732S). Primary antibody membranes were washed thrice with TBST containing 2% (w/v) BSA, after which they were incubated with Goat Anti-Mouse IgG-HRP (1:5000) (SouthernBiotech, 1010–05) or Goat Anti-Rabbit IgG-HRP (1:5000) (SouthernBiotech, 4010–05) in 2% (w/v) BSA-TBST and washed thrice with TBST. All membranes were subsequently developed with WesternBright ECL (Advansta) and imaged on ImageQuant LAS 4000 (GE HealthCare). (ii) Ptx holotoxin experiments: To first activate the Ptx holotoxin, *i.e.*, to reduce the autoinhibitory Cys41-Cys202 disulfide bond, 0.2 or 1 μg of Pertussis Holotoxin (Biotrend, LL-179A) was coincubated with 20 mM DTT for 30 min at room temperature. Next, 1 μM of biotinylated NAD+ (Trevigen, 4670–500–01) was added with or without 2 μM rGαi substrate followed by incubation for 40 min at room temperature with shaking at 300 rpm. Reactions were conducted in 100 mM Hepes (pH 7.5), 500 mM NaCl, 10% (w/v) glycerol, and 2 mM DTT with a final volume of 50 μl. Reactions were stopped by addition of Laemmli loading dye to 1x and heating for 10 min at 95 °C. The samples were run on SDS-PAGE and transferred to nitrocellulose membranes, followed by analysis for proteins and ADP-ribose conjugants as described above. Additionally, the anti-PT monoclonal antibody (S1) (NIBSC, 99/512) (1:1000) for Pertussis S1-subunit was used using membrane blocking and primary antibody washes with 5% (w/v) nonfat milk in TBST.

### Mass spectrometry analysis of ADP-ribose conjugates

(i) Mass spectrometry sample preparation: The wt rPtxS1 (10 μM) was incubated at room temperature for 3 h in a 100 μl reaction volume [100 mM Hepes (pH 7.5), 500 mM NaCl, 10% glycerol, and 2 mM DTT] under the following conditions (1): rPtxS1 (10 μM) (2), rPtxS1 (10 μM), and 10 μM NAD+ (Sigma-Aldrich, N1511), and (3) rPtxS1 (10 μM), rGαi (10 μM) and 10 μM NAD+ (Sigma-Aldrich, N1511). For denaturation and cysteine reduction, the proteins were incubated in 3 M urea (Sigma-Aldrich, U4884) and 0.01 M of DTT (Sigma-Aldrich, 43819) for 1 h at room temperature. Then, samples were alkylated with 0.05 M of iodoacetamide (Sigma-Aldrich, I6125) at room temperature for 1 h in the dark (iodoacetamide is light-sensitive). A 10K Amicon Ultra Centrifugal Filter (Merk, UFC501008) was used to exchange the buffer to 25 mM ammonium bicarbonate (Sigma-Aldrich, 09830). Proteins were digested with addition of trypsin [1:30 (w:w) trypsin to protein, Promega, V5117] overnight at 37 °C. The 10K Amicon Ultra Centrifugal Filter was used to remove undigested proteins. The eluted peptides were dried in a vacuum centrifuge system (Savant Speedvac Concentrator SVC-100) and stored at −20 °C. (ii) Mass spectrometry runs: Mass spectrometry analyses were performed at the Turku Proteomics Facility supported by Biocenter Finland. The LC-ESI-MS/MS analyses were performed using a nanoflow HPLC system (Easy-nLC1200, Thermo Fisher Scientific) coupled with an Orbitrap Fusion Lumos mass spectrometer. The peptides were first loaded on a trap column (100 μm ID x 2 cm) and subsequently separated inline on an analytical column (75 μm ID x 15 cm). Both columns were in-house packed with ReproSil-Pur 3 μm 120 Å C18-AQ bulk media (Dr Maisch HPLC GmbH, Ammerbuch-Entringen, Germany). The mobile phase consisted of H_2_O and 0.1% formic acid (solvent A), and acetonitrile/H_2_O (80:20 (v/v)) and 0.1% formic acid (solvent B). Peptides were eluted with the following 30 min gradient of solvent B: from 5% to 35% in 20 min, from 35% to 100% in 5 min followed by a wash for 5 min at 100%. The flow rate was 300 nl/min. Mass spectrometry analysis was carried out in a data-dependent acquisition mode with a cycle time set to 3 s. MS1 spectra were acquired with a resolution of 120,000, a scan range of m/z 350–m/z 2000, an automatic gain control target value of 200,000 and a maximum injection time of 50 ms. Precursor ions were at first fragmented with high-energy collisional dissociation (HCD) fragmentation using a quadrupole isolation window of m/z 1.6, a normalized collision energy of 38%, an orbitrap resolution of 15,000, and a maximum injection time of 22 ms. If at least three ADP-ribose fragment ions (m/z 136.06, m/z 250.09, m/z 348.07, or m/z 428.04) were detected, second HCD fragmentation, and EThcD fragmentation were triggered for ADP-ribosylated peptide candidates. Precursors for the first HCD fragmentation were filtered using a monoisotopic peak selection set to peptide, a charge state filter allowing only charge states 2 to 6, a minimum intensity threshold of 50,000 and a dynamic exclusion time of 15 s. Spectra in second HCD fragmentation and in EThcD fragmentation were acquired with a quadrupole isolation window set to m/z 1.6, an orbitrap resolution of 30,000, an automatic gain control target of 500,000, a maximum injection time of 54 ms and a dynamic exclusion time of 15 s. A normalized collision energy of 30% was used in second HCD fragmentation and a supplemental activation energy of 25% was used in EThcD fragmentation. A number of dependent scans was set to 10. The mass spectrometry proteomics data have been deposited to the ProteomeXchange Consortium (https://www.proteomexchange.org) *via* the PRIDE (43) partner repository with the dataset identifier PXD050185. (iii) Mass spectrometry data analysis. Data analysis was performed by using Proteome Discoverer (Thermo Fisher Scientific, version 2.5) connected to the Mascot search engine (Matrix Science, London, UK; version 2.8.2). Peptide and protein identifications were performed by the Mascot search engine. The data were searched against the pertussis toxin subunit 1 protein sequence (*Bordetella pertussis*, UniProt SwissProt accession P04977) and the UniProt SwissProt human database (UniProt release 2023_01). A fragment ion mass tolerance of 0.02 Da and a parent ion mass tolerance of 10 ppm were used. Enzyme specificity was set to trypsin, allowing up to two missed cleavages. Carbamidomethylation of cysteine was specified as a fixed modification, and oxidation of methionine and ADP-ribosylation were set as variable modifications. Arginine, asparagine, aspartic acid, cysteine, glutamic acid, glutamine, histidine, lysine, serine, threonine, and tyrosine residues were selected as potential ADP-ribose acceptor sites. The neutral losses from the ADP-ribose modification equal to 249.09 Da, 347.06 Da, and 583.08 Da were used for scoring in HCD fragment ion spectra. The ADP-ribose fragment ions at m/z 136.06, 250.09, 348.07, and 428.04 were excluded from scoring in HCD and in EThcD spectra (44). The Mascot search engine calculated ADP-ribosylation site localization probabilities. Peptide spectrum matches featuring site localization probability above 75% and Mascot expectation value below 0.05 were included in the final results. In house written R scripts were used to extract data from the Mascot reports.

### Biocatalytic production of BaAD and analogs

(i) Reactions: The reactions (10–25 μl) contained 25 to 400 μM rPtxS1 wt or mutants (C41S, C41G, and Q127D/E129D), 2 to 50 mM 3-AB (Princeton Bio, PBMR044728 or Selleckchem, S1132, both dissolved in water)/3-AB analogs (Molport, see Table S1 and Fig. S5, all dissolved in water) and 2 to 20 mM NAD+ (Sigma-Aldrich, N3014, dissolved in water) in 100 mM Hepes (pH 7.5), 500 mM NaCl, 10% glycerol, and 2 mM DTT. First, rPtxS1 and 3-AB/3-AB analogs were combined in the reaction buffer and incubated for 30 min at room temperature. Next, NAD+ was added and the reactions were incubated for 3 to 72 h at room temperature. The reactions were stored frozen at −80 °C until HPLC and LC-MS analysis. (ii) Yield estimation with the AUC method. The concentrations of the NAD+ analogs were estimated by comparing the AUC of the analogs with that of a known concentration of NAD+ from the HPLC chromatograms. The AUC of NAD+ was measured without the PtxS1 to establish a reference. The concentrations of the NAD+ analogs were then calculated based on the proportional relationship between the AUC and concentration. (iii) Yield estimation with the calibration curve method. A calibration curve was prepared using standard concentrations of NAD+ and measuring absorbance at 260 nm using a UV-Vis Nano Drop spectrophotometer. The concentration of the NAD+ analogs was subsequently estimated by interpolating its absorbance on the NAD+ calibration curve. (iv) Hydrolytic stability estimation. To determine the hydrolytic stability of BaAD, IiaAD, and IqaAD, the nucleotides were first biocatalytically produced for 72 h at room temperature in 25 μl reactions (80 μM wt rPtxS1, 20 mM NAD+, and 20 mM 3-AB/3-AB analogs). Subsequently, 100 μl of either 0.1 M NaOH or MQ H_2_O was added. The reactions were centrifuged (9270g, 10 min, room temperature) through a 10K centrifugal filter unit (Pall Nanosep 10K Ω Centrifugal Devices, OD010C34) to remove the enzyme. The reactions were further incubated for 72 h at room temperature, and stored frozen at −80 °C until HPLC analysis.

### HPLC and MS analysis of BaAD and analogs

(i) Sample preparation. The reactions were diluted 4 to 10-fold with water, and after careful mixing they were incubated for 15 min at room temperature. The reactions were centrifuged (9270g, 10 min, room temperature) through a 10K centrifugal filter unit (Pall Nanosep 10K Omega Centrifugal Devices, OD010C34) to remove the enzyme. The filtrate was collected and injected into HPLC in varying volumes between 10 and 50 μl. Also, the BaAD content in the centricon-trapped proteins was analyzed. Accordingly, the proteins were recovered to a fresh tube from the centricon filters by adding 100 μl of H_2_O. The solutions were heated for 5 min at 75 °C and centrifuged through a 10K centrifugal filter to remove the denatured enzyme, as described above. Filtrate was collected and injected into HPLC (20–100 μl). (ii) HPLC analysis—method A. The reference compounds (NAD+, Sigma-Aldrich, N3014; 3-AB, Selleckchem, S1132; nicotinamide, Sigma-Aldrich, N0636; ADP-ribose, Sigma-Aldrich, A0752) and reaction filtrates were analyzed by reverse-phase HPLC using a C18 column (150 mm × 4.6 mm x 5 *μ*m, SupelcoAnalytical) with 1 ml/min flow rate with the following solvents: solvent A, 0.2 M triethylammonium acetate (pH 7.0) in H_2_O; solvent B, H_2_O, solvent C, acetonitrile (MeCN). The method involves a gradual increase in the solvent C percentage from 0 to 30% over a duration of 15 min, while simultaneously keeping the solvent A percentage constant at 20%. (iii) HPLC analysis*—*method B. The reference compounds (as explained above) and reaction filtrates were analyzed by Agilent 1200 series reverse-phase HPLC using a C18 column (Waters SunFire C18, 150 mm × 2.1 mm x 3.5 *μ*m column, 186002535) by isocratic elution using 10 mM (NH_4_)H_2_PO_4_ (pH 5.5) and acetonitrile (97.5:2.5) mobile phase with 0.4 to 0.5 ml/min flow rate (4, 45, 46). The detection was carried out by photo diode array detector with detection wavelength set at 260 nm. The data were generated and analyzed by Agilent ChemStation software (https://agilent.com) and further processed in GraphPad prism. To confirm the absence of any coeluting impurities, peak purity determination was carried out for all eluting components by taking five UV spectra per peak. (iv) LC-MS analysis: To perform LC-MS analysis, peaks were collected (HPLC analysis—method B), vacuum dried using Savant integrated SpeedVac concentrator system, and dissolved in LC-MS grade water. LC-MS analyses were performed using Waters Acquity RDa system with a C18 column (30 mm × 4.6 mm × 3.5 *μ*m, 130 Å, XBridge BEH). The mobile phase (0.8 ml/min flow rate) in gradient elution mode with the following solvents combinations was used: solvent A, H_2_O + 0.1% formic acid; solvent B MeOH + 0.1% formic acid. At zero minute the ratio of solvent A and B was kept to 95:5, at 2.2 min the ratio changed to 100% solvent B which was kept constant up to 4.6 min. The solvent composition was brought to initial condition at 4.8 min and was kept constant up to 5.0 min. The detection was carried out using ACQUITY RDa Detector in small molecule positive ion mode using full scan with fragmentation mode. The cone voltage was set at 30V while fragmentation cone voltage was set at 60 to 120V. The capillary voltage was set to default value of 1.5 kV with desolvation temperature of 550 °C.

### Fluorescence analysis

The fluorescence analysis of BaAD and its two analogs dissolved in MQ water was performed using NADH (Sigma-Aldrich, N8129) dissolved in MQ water as a reference compound. The fluorescence measurements were performed initially using VICTOR Nivo multimode plate reader (Revvity), with excitation and emission wavelengths set at 355 (+/40 nm) and 450 nm ( ± 10 nm), respectively. The measurements were performed in a 96-well black plate with a flat bottom (Greiner Bio-One, 655209). The fluorescence spectral analysis was performed using Spark 20M from Tecan Life Sciences from the same plate as the above measurements. Excitation spectra (230–400 nm) were recorded with 5 nm slit, 430 nm emission with 20 nm slit and 40 μs integration time. Emission spectra (360–600 nm) were monitored with 5 nm slit, 330 nm excitation with 10 nm slit, and integration time of 40 μs. All the reported data are based on MQ water-substracted values.

## Data availability

The mass spectrometry proteomics data have been deposited to the ProteomeXchange Consortium (https://www.proteomexchange.org) *via* the PRIDE (43) partner repository with the dataset identifier PXD050185. All the other data are contained within the manuscript.

## Supporting information

This article contains supporting information.

## Conflict of interest

The authors declare that they have no conflicts of interest with the contents of this article.
