# Supplementary material for: ADP-ribosyltransferase-based biocatalysis of nonhydrolyzable NAD+ analogs
Source: J Biol Chem. 2024 Dec 18;301(1):108106. doi: 10.1016/j.jbc.2024.108106 (PMC11786771; doi:10.1016/j.jbc.2024.108106)
Supplement: Supplementary Figures [file mmc1.pdf]

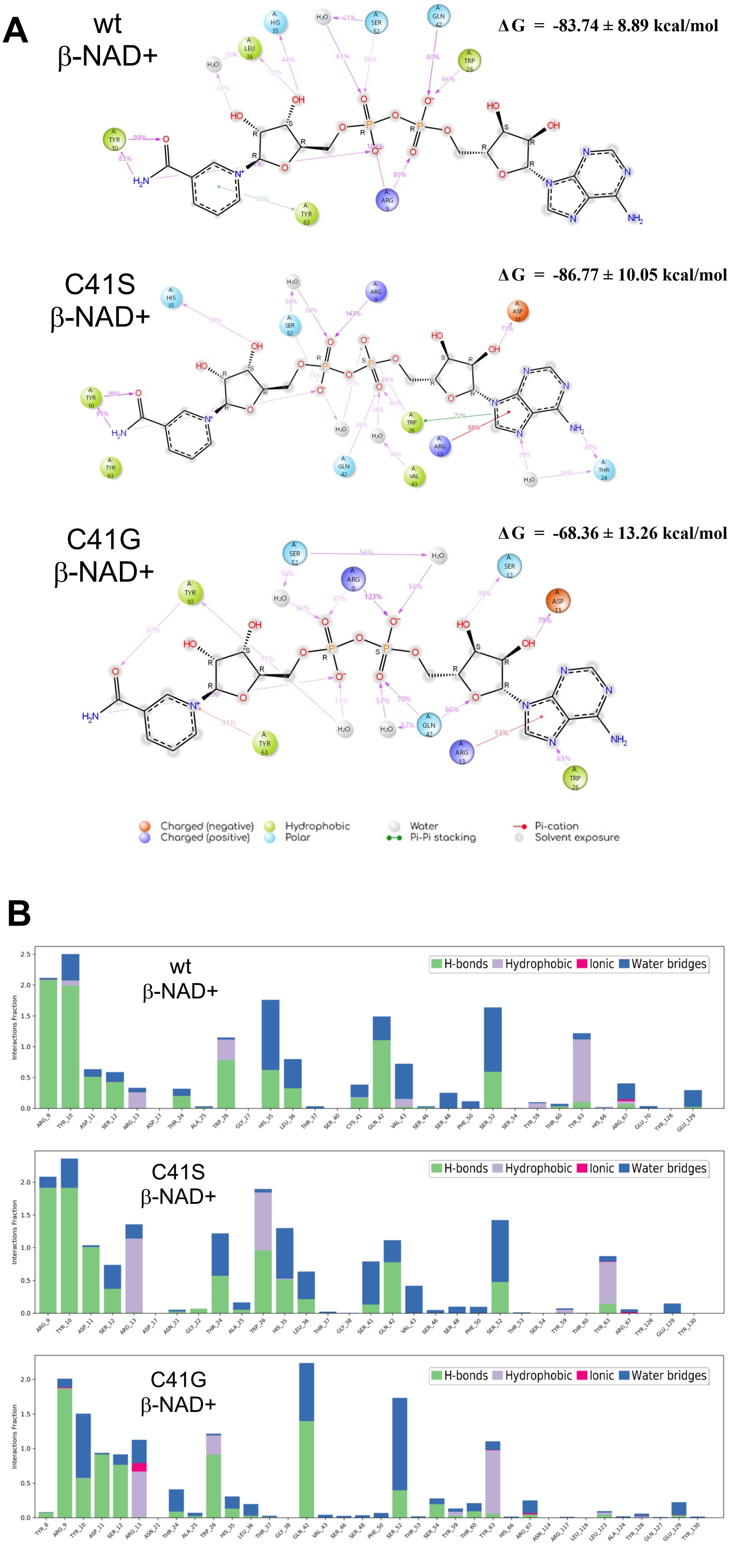
A
wt
β-NAD+
ΔG = -83.74 ± 8.89 kcal/mol
C41S
β-NAD+
ΔG = -86.77 ± 10.05 kcal/mol
C41G
β-NAD+
ΔG = -68.36 ± 13.26 kcal/mol
Charged (negative)
Charged (positive)
Hydrophobic
Polar
Water
Pi-Pi stacking
Pi-cation
Solvent exposure
B
wt
β-NAD+
C41S
β-NAD+
C41G
β-NAD+
H-bonds
Hydrophobic
Ionic
Water bridges
Interactions Fraction

Figure S1.

Figure S1. ***In silico*** **characterization of a series of PtxS1 mutants. A)** The interactions of wt rPtxS1 and its two *in silico* mutants C41S and C41G with co-crystallised β-NAD+ (PDB ID:7SKY), over the course of a 500-ns MD simulation (% denotes the duration of an interaction from the total simulation time). Only interactions that lasted for more than 30% of the simulation time are considered. **B)** Ligand-receptor interaction histogram showing interactions of wt rPtxS1 and *in silico* C41S and C41G mutants with co-crystallised β-NAD+ (NAD+ structure of rPtxS1, PDB 7SKY). The stacked bar charts are normalized over the course of a 500-ns MD simulation trajectory. Therefore, the interactions fraction value denotes how long the interaction lasted with respect to the length of the total simulation. If the interactions fraction exceeds 1.0 (100% of the simulation time), the respective residue forms more than one interaction with the ligand.

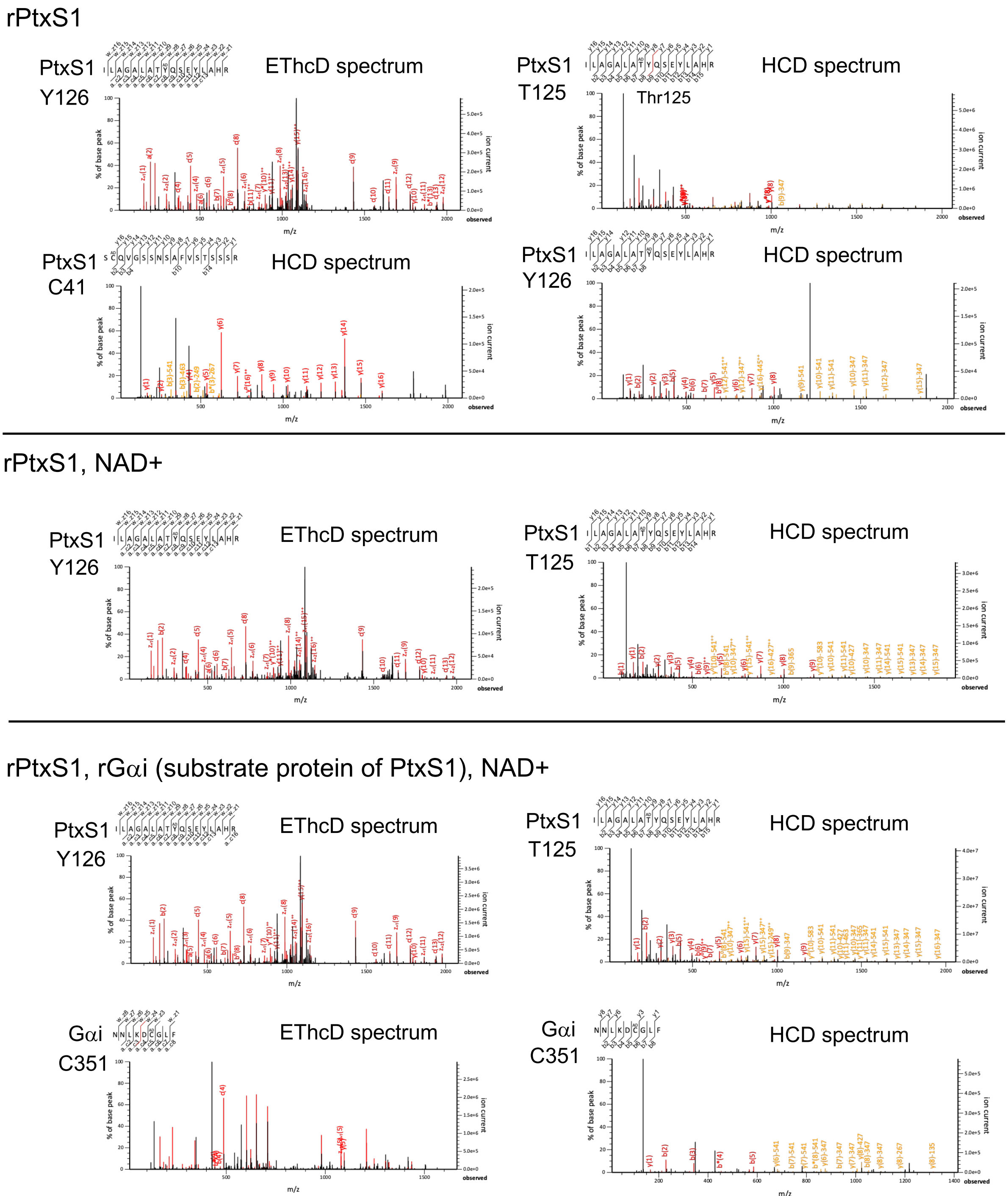
rPtxS1
PtxS1 Y126
EThcD spectrum
PtxS1 T125
HCD spectrum
Thr125
PtxS1 C41
HCD spectrum
PtxS1 Y126
HCD spectrum
rPtxS1, NAD+
PtxS1 Y126
EThcD spectrum
PtxS1 T125
HCD spectrum
rPtxS1, rGαi (substrate protein of PtxS1), NAD+
PtxS1 Y126
EThcD spectrum
PtxS1 T125
HCD spectrum
Gαi C351
EThcD spectrum
Gαi C351
HCD spectrum
% of base peak
ion current
m/z
observed

Figure S2.

Figure S2. Examples of the spectra of ADP-ribosylated peptides. The recombinant PtxS1 (rPtxS1) was either directly subjected to mass spectrometry-based analysis, or it was first incubated with NAD+, or it was first co-incubated with NAD+ and recombinant Gαi (rGαi). The ADP-ribosylated peptide identification was achieved with two different fragmentation techniques, i.e. electron-transfer/higher-energy collision dissociation (EthcD) and higher-energy C-trap dissociation (HCD) (see methods section for details). In EthcD spectra, red peaks denote the peptides identified with or without ADP-ribose and plack peaks denote the unidentified peptides. In HCD spectra, yellow peaks denote the peptides identified with ADP-ribose, red peaks denote the peptides identified without ADP-ribose, and plack peaks denote the unidentified peptides. Residue numbering refers to PtxS1 (P04977, where position number 1 is aspartic acid DDPP…), and Gαi (P63096). The key data reliability metrics of all the identified ADP-ribosylated peptides are shown in the Supplementary data file 1.

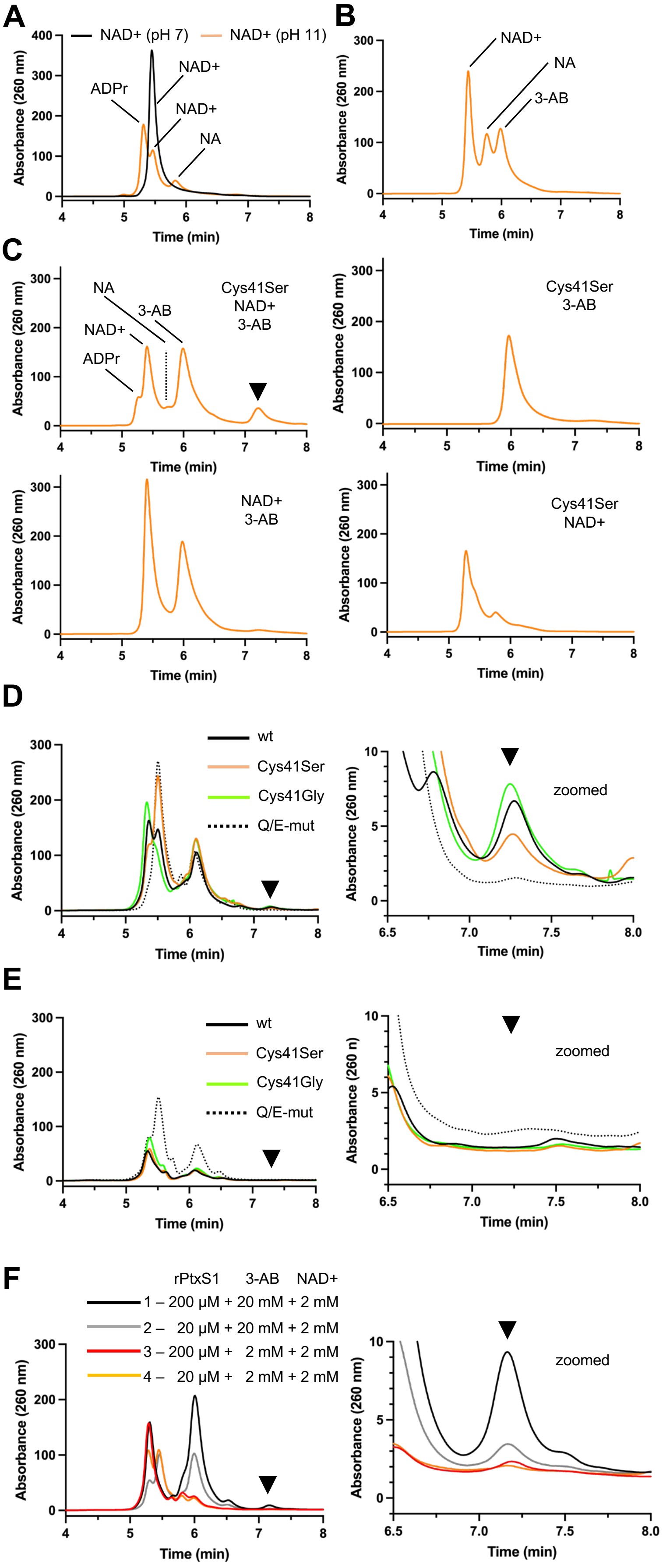
A
NAD+ (pH 7)
NAD+ (pH 11)
ADPr
NAD+
NAD+
NA
Absorbance (260 nm)
Time (min)
B
NAD+
NA
3-AB
Absorbance (260 nm)
Time (min)
C
NA
3-AB
NAD+
ADPr
Cys41Ser
NAD+
3-AB
Absorbance (260 nm)
Time (min)
Cys41Ser
3-AB
NAD+
3-AB
Cys41Ser
NAD+
D
wt
Cys41Ser
Cys41Gly
Q/E-mut
zoomed
Absorbance (260 nm)
Time (min)
E
wt
Cys41Ser
Cys41Gly
Q/E-mut
zoomed
Absorbance (260 n)
Time (min)
F
rPtxS1 3-AB NAD+
1 – 200 µM + 20 mM + 2 mM
2 – 20 µM + 20 mM + 2 mM
3 – 200 µM + 2 mM + 2 mM
4 – 20 µM + 2 mM + 2 mM
zoomed
Absorbance (260 nm)
Time (min)

Figure S3.

Figure S3. HPLC-based analysis of PtxS1-catalyzed BaAD formation. A) HPLC trace of NAD+ incubated at neutral (water, pH 7) or highly basic aqueous solution (0.1 mM NaOH, pH 11) for 4 hours at room temperature. High pH induces hydrolysis of NAD+, upon which nicotinamide (NA), and ADP-ribose become visible in the HPLC trace. **B)** HPLC trace of co-injected NAD+, NA and 3-AB. **C)** HPLC trace of IMAC-purified C41S mutant (200 μM) reaction after incubation with different reaction components for 3 h at RT, and filtration with a 10 kDa cutoff centricon. The arrow head marks the position of a peak at around 7.25 min retention time that only appeared when the mutant protein was incubated together with 3-AB (20 mM) and NAD+ (4 mM). **D-E)** Analysis of BaAD formation by a series of rPtxS1 mutants and wt (200 μM), as purified with IMAC, after incubation with different reaction components for 3 h at RT (20 mM 3-AB, 2 mM NAD+). The BaAD peak highlighted with an arrowhead, i.e., the peak at around 7.25 min retention time, is zoomed on the right. Data in D panels comes from samples after filtration with 10 kDa cutoff centricon. Data in E panels comes from samples that were retrieved from the 10 kDa cutoff centricon filters, heated to release any protein-bound nucleotides, and subsequently passed through 10 kDa cutoff centricon filters. Additional controls are shown in Fig. S4. **F)** HPLC trace of SEC-purified wt rPtxS1 (200 μM), reaction after incubation under different reaction conditions for 3 h at RT, and filtration with a 10 kDa cutoff centricon. The BaAD peak highlighted with an arrowhead, i.e., the peak at around 7.25 min retention time, is zoomed on the right. All the above HPLC runs were executed with the HPLC method A.

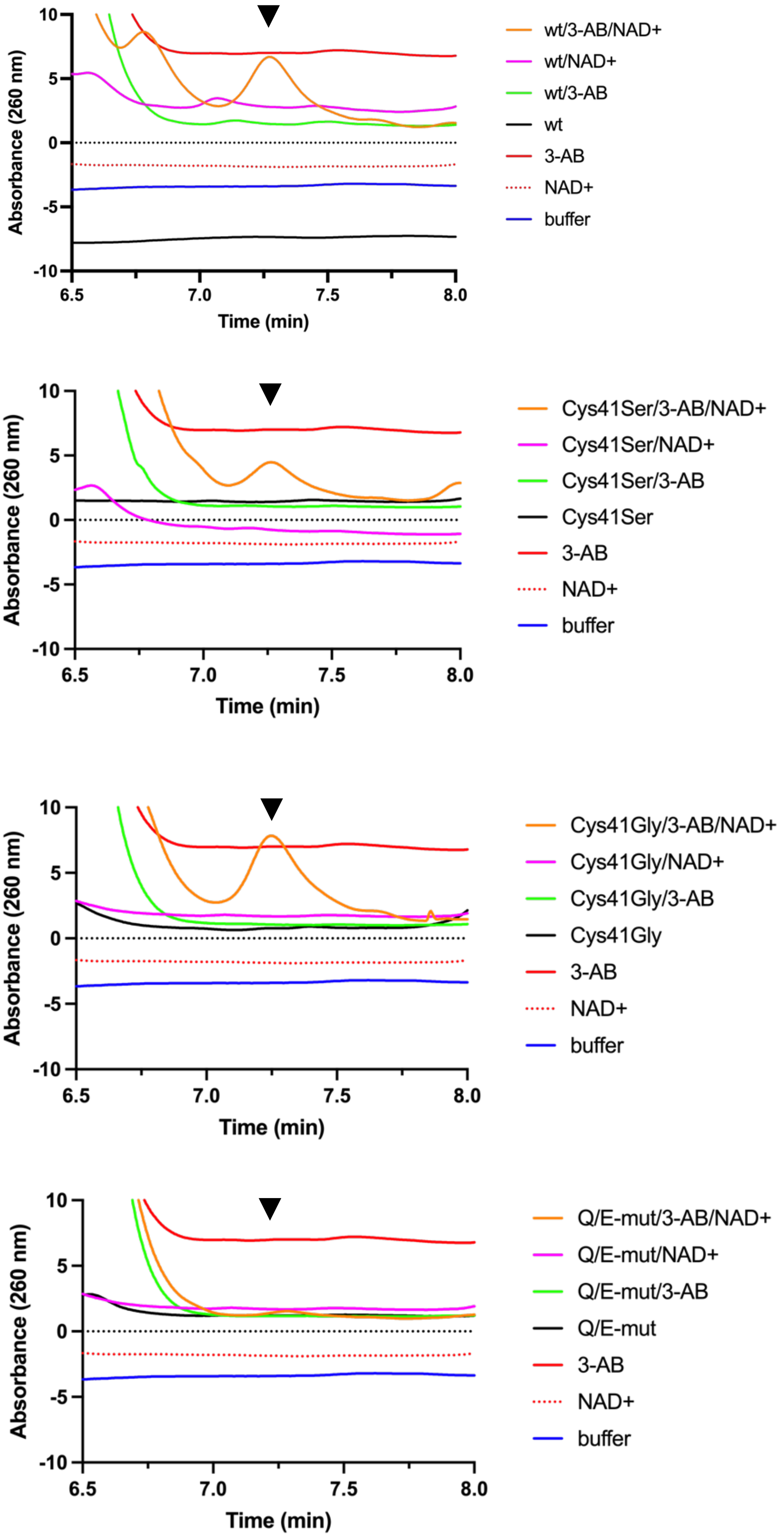

Absorbance (260 nm)
Time (min)
wt/3-AB/NAD+
wt/NAD+
wt/3-AB
wt
3-AB
NAD+
buffer
Cys41Ser/3-AB/NAD+
Cys41Ser/NAD+
Cys41Ser/3-AB
Cys41Ser
Cys41Gly/3-AB/NAD+
Cys41Gly/NAD+
Cys41Gly/3-AB
Cys41Gly
Q/E-mut/3-AB/NAD+
Q/E-mut/NAD+
Q/E-mut/3-AB
Q/E-mut

Figure S4.

Figure S4. HPLC-based analysis of PtxS1-catalyzed BaAD formation. HPLC traces of IMAC-purified wt and mutant rPtxS1 (200 µM) reactions after incubation with different reaction components for 3 h at RT, and filtration with a 10 kDa cutoff centricon. The arrow head marks the position of a peak that only appeared when the proteins were incubated together with 3-AB (20 mM) and NAD+ (4 mM). This experiment is the same as shown in Fig. S3D, but the HPLC traces of all the analyzed reaction conditions are shown here. All the above HPLC runs were executed with the HPLC method A.

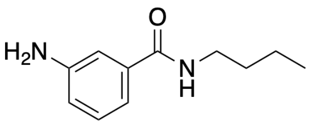

3-amino-N-butylbenzamide
CAS - 6837-99-6

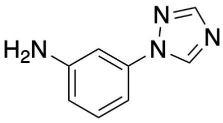

3-(1H-1,2,4-triazol-1-yl)aniline
CAS - 176032-78-3

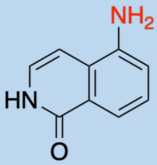

5-amino-1,2-dihydroisoquinolin-1-one
CAS - 93117-08-9

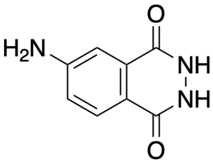

6-amino-1,2,3,4-tetrahydrophthalazine-1,4-dione
CAS - 3682-14-2

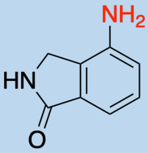

4-amino-2,3-dihydro-1H-isoindol-1-one
CAS - 366452-98-4

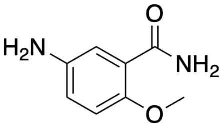

5-amino-2-methoxybenzamide
CAS - 22961-58-6

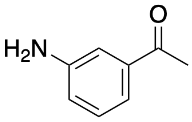

1-(3-aminophenyl)ethan-1-one
CAS - 99-03-6

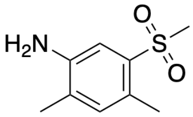

5-methanesulfonyl-2,4-dimethylaniline
CAS - 849035-63-8

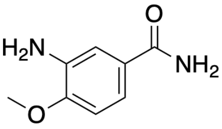

3-amino-4-methoxybenzamide
CAS - 17481-27-5

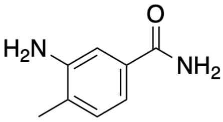

3-amino-4-methylbenzamide
CAS - 19406-86-1

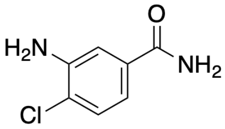

3-amino-4-chlorobenzamide
CAS - 19694-10-1

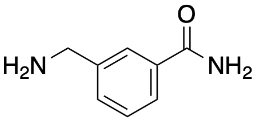

3-(aminomethyl)benzamide
CAS - 102562-86-7

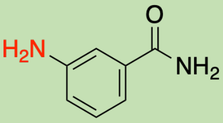

3-aminobenzamide
CAS - 3544-24-9

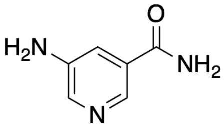

5-aminopyridine-3-carboxamide
CAS - 60524-14-3

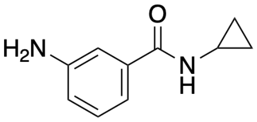

3-amino-N-cyclopropylbenzamide
CAS - 871673-24-4

Figure S5.

Figure S5. Structures of 3-AB analogs predicted by *in silico* molecular orbital theorem assisted modeling exercise. The modeling was executed to identify 3-AB analogs, which would have the capacity to insert into the nicotinamide pocket of PtxS1, and also would have the amino group-associated nucleophilic power to attack the PtxS1-formed oxocarbenium cation. The 3-AB analogs highlighted in blue yielded products by PtxS1-mediated catalysis.

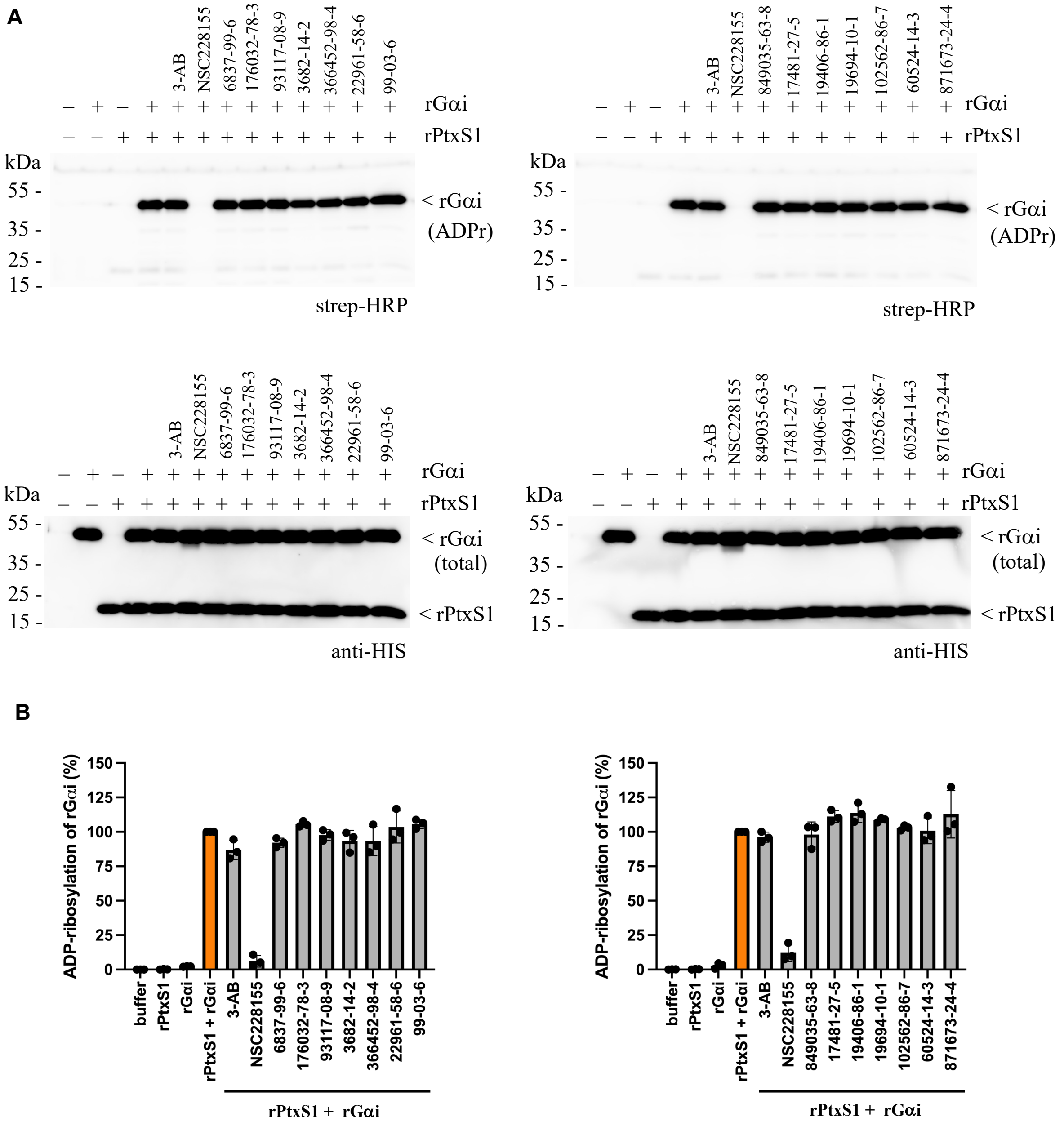
A
B
rGαi
rPtxS1
kDa
< rGαi (ADPr)
strep-HRP
< rGαi (total)
< rPtxS1
anti-HIS
3-AB
NSC228155
6837-99-6
176032-78-3
93117-08-9
3682-14-2
366452-98-4
22961-58-6
99-03-6
849035-63-8
17481-27-5
19406-86-1
19694-10-1
102562-86-7
60524-14-3
871673-24-4
ADP-ribosylation of rGαi (%)
buffer
rPtxS1
rGαi
rPtxS1 + rGαi
rPtxS1 + rGαi

Figure S6.

Figure S6. Effect of small molecular weight compounds on the Gαi specific ADP-ribosylation activity of PtxS1. *In vitro* ADP-ribosylation assay for rPtxS1 (200 nM) with NAD+-biotin (2 μM) and the rGαi (1 μM) substrate protein of PtxS1. The 3-AB was analyzed at 1 mM and the rest of the small molecular weight compounds at 200 μM concentrations. NSC228155 is a previously identified inhibitor of the Gαi specific ADP-ribosylation activity of PtxS1 (Ashok et al 2020, https://doi.org/10.1021/acsinfecdis.9b00412). **A)** Protein-conjugated biotin-ADP-ribose (ADPr) was detected with streptavidin-HRP. The same samples were analyzed in two parallel membranes (streptavidin-HRP and anti-HIS). **B)** Quantitation of the compound effect based on the densitometric analysis of rPtxS1-mediated rGαi ADP-ribosylation. The quantitation is based on strep-HRP blot densitometric values of rGαi areas from three independent experiment (means +/- SD). Blot data of one representative experiment are shown in sub-panel A.

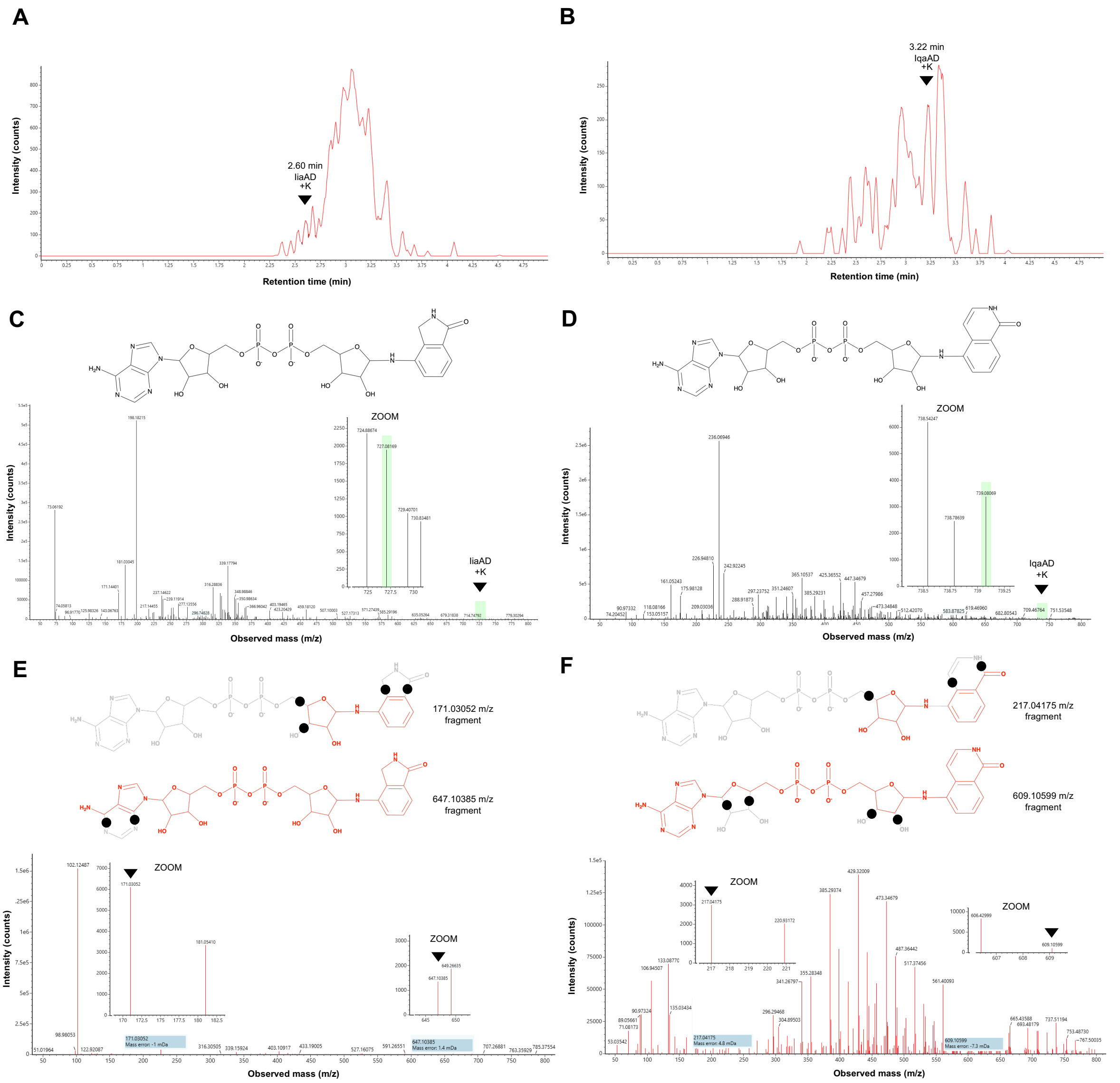

G

adduct	neutral molar mass (g/mol)	m/z	retention time (min)
+Na	688.1240	711.1132	3.20
-K	688.1185	727.0817	2.60

expected neutral molar mass of liaAD = 688.11695 g/mol

H

adduct	neutral molar mass (g/mol)	m/z	retention time (min)
-K	700.1175	739.0807	3.22

expected neutral molar mass of IqaAD = 700.11695 g/mol

Figure S7.

Figure S7. Mass spectrometry-based analysis of PtxS1-catalyzed formation of BaAD analogs. A-B) Chromatograms of LC-MS analyses of IiaAD and IqaAD. **C-D)** Mass spectra with 30V cone voltage (+K adducts of IiaAD and IqaAD). **E-F)** Mass spectra with fragmentation 60-120 V cone voltage (+K adducts of IiaAD and IqaAD). Two example fragments are shown, which led into identification of IiaAD and IqaAD. **G-H)** Different adducts of IiaAD and IqaAD detected in the MS runs.

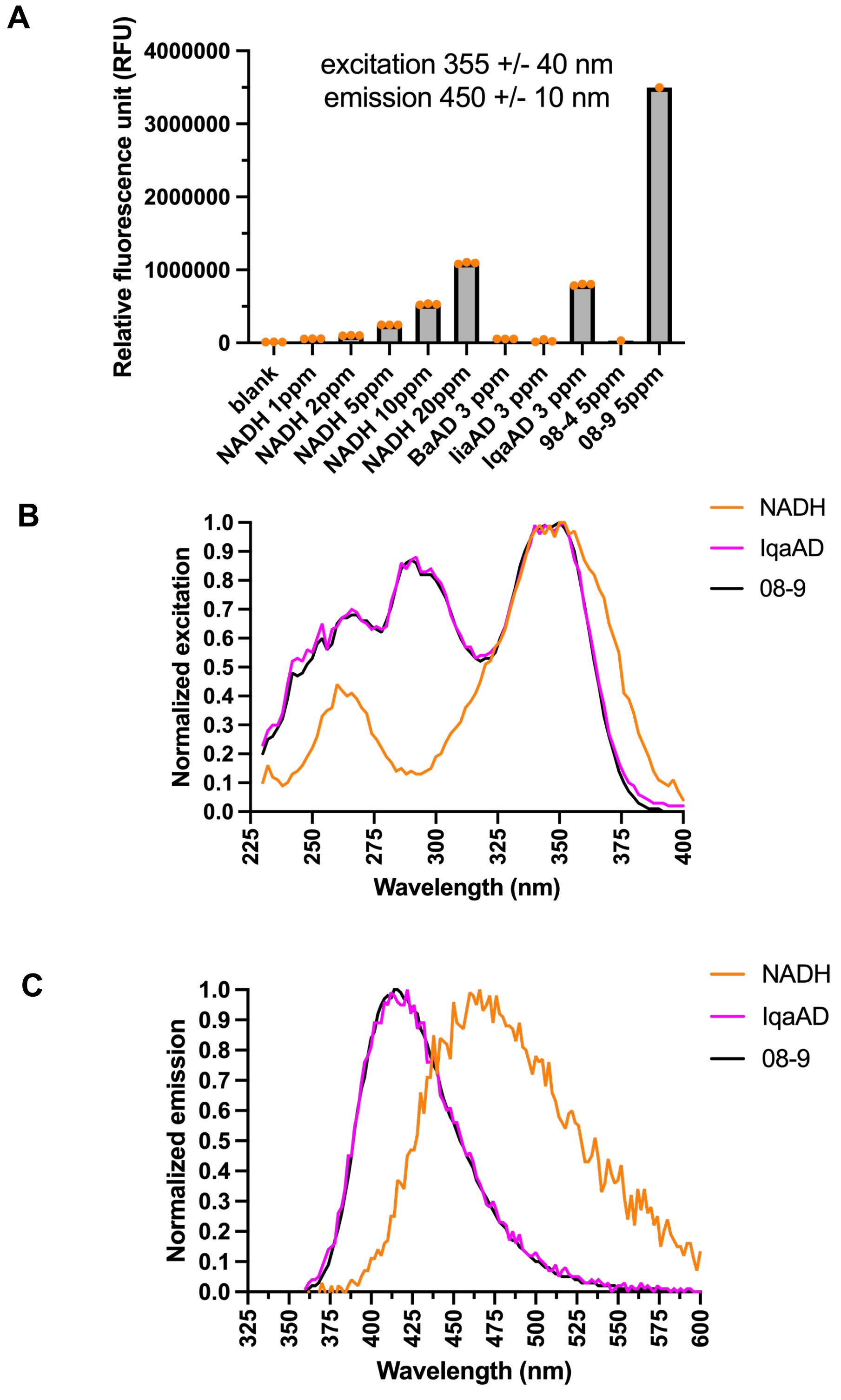
A
Relative fluorescence unit (RFU)
4000000
3000000
2000000
1000000
0
excitation 355 +/- 40 nm
emission 450 +/- 10 nm
blank
NADH 1ppm
NADH 2ppm
NADH 5ppm
NADH 10ppm
NADH 20ppm
BaAD 3 ppm
liaAD 3 ppm
IqaAD 3 ppm
98-4 5ppm
08-9 5ppm
B
Normalized excitation
Wavelength (nm)
NADH
IqaAD
08-9
C
Normalized emission
Wavelength (nm)
NADH
IqaAD
08-9

Figure S8.

Figure S8. Fluorescence properties of BaAD and its analogs. A) Fluorescence emission intensities of BaAD and its analogs at 450 nm (+/- 10 nm) as measured with 355 nm excitation (+/- 40 nm). The fluorescent NADH is measured as a control. **B)** Fluorescence excitation and emission spectra of BaAD and its analogs. The fluorescent NADH is measured as a control. Excitation spectra (230–400 nm) were recorded with 430 nm emission, and the emission spectra (360–600 nm) were recorded with 330 nm excitation. The spectra are based on normalized values, i.e., the maximum recorded value for a given compound has been set as 1.0.

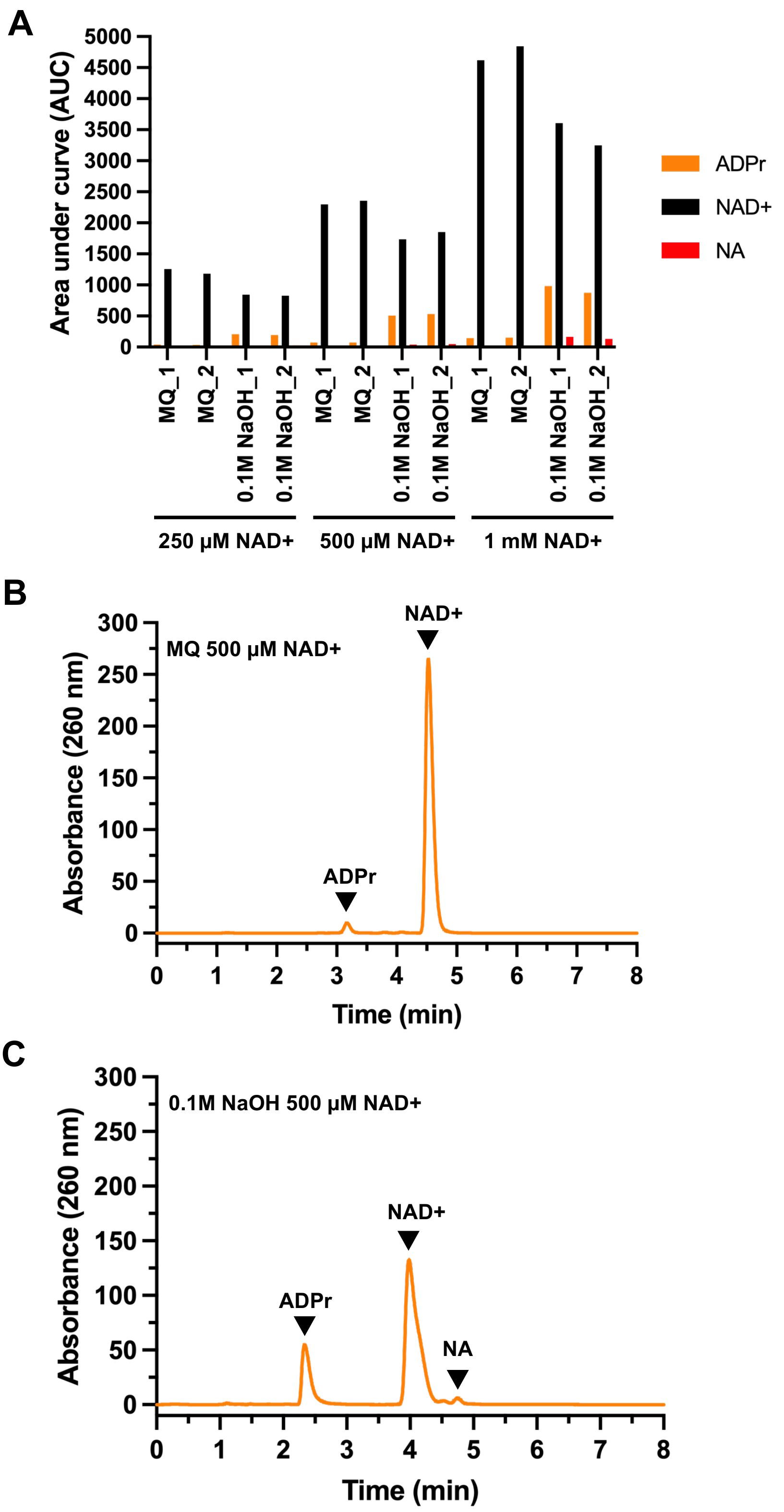
A
Area under curve (AUC)
5000
4500
4000
3500
3000
2500
2000
1500
1000
500
0
MQ_1
MQ_2
0.1M NaOH_1
0.1M NaOH_2
250 µM NAD+
500 µM NAD+
1 mM NAD+
ADPr
NAD+
NA
B
Absorbance (260 nm)
MQ 500 µM NAD+
NAD+
ADPr
300
250
200
150
100
50
0
0 1 2 3 4 5 6 7 8
Time (min)
C
Absorbance (260 nm)
0.1M NaOH 500 µM NAD+
NAD+
ADPr
NA
300
250
200
150
100
50
0
0 1 2 3 4 5 6 7 8
Time (min)

Figure S9.

Figure S9. HPLC-based analysis of high pH-induced hydrolysis of NAD+. A) NAD+ was incubated at room temperature for 72 hours in duplicate in MQ-water or in 0.1 M NaOH. Each reaction was injected twice to the HPLC system. The peak profiles of the HPLC chromatograms [NAD+, ADP-ribose (ADPr) and nicotinamide (NA)] were converted to area under curve (AUC) values. The reported AUC values refer to the average AUC values of the two independent HPLC injections. **B-C)** Representative single injection examples of the HPLC chromatograms with 500 μM NAD+.

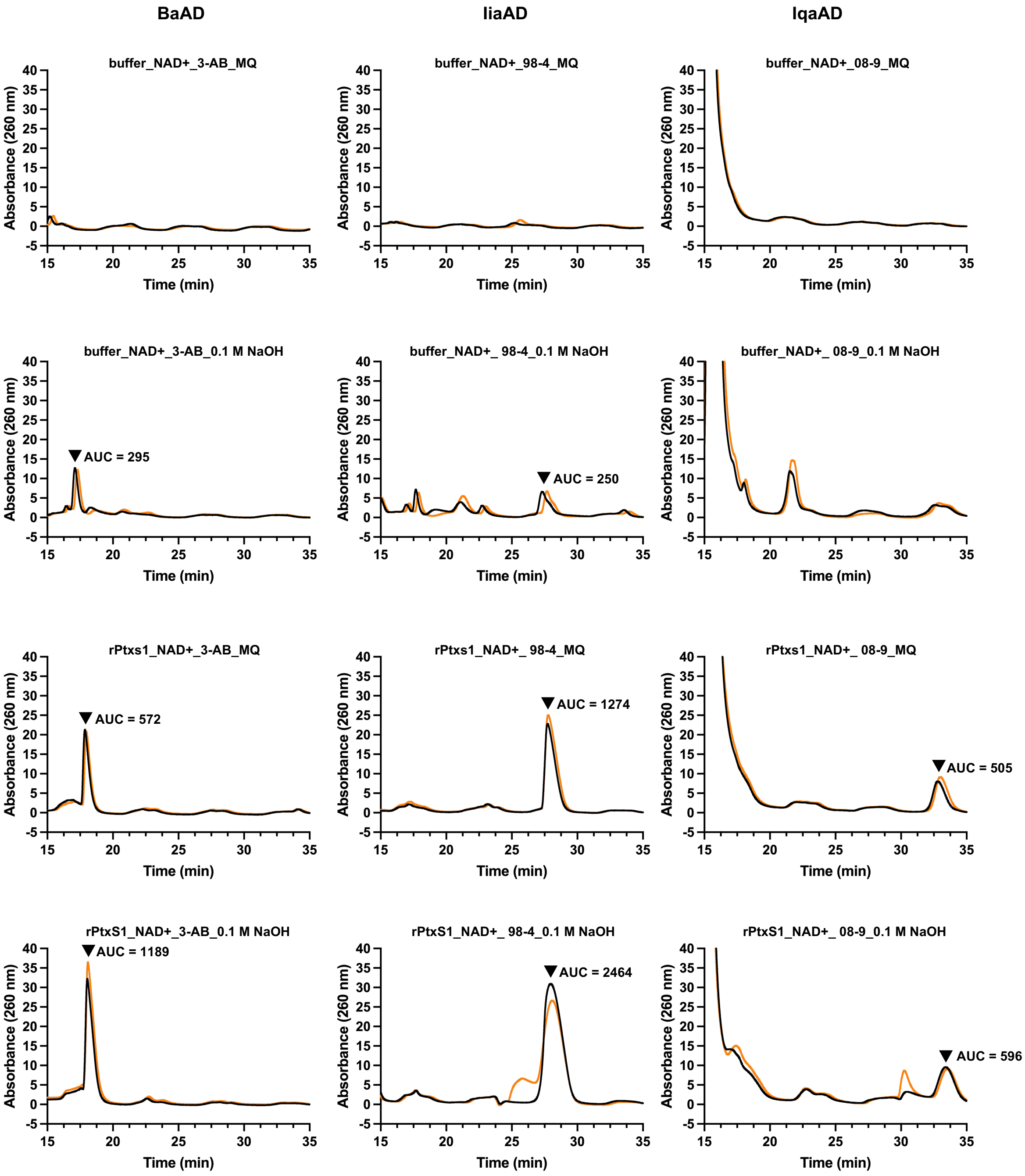
BaAD
IiaAD
IqaAD
buffer_NAD+_3-AB_MQ
buffer_NAD+_98-4_MQ
buffer_NAD+_08-9_MQ
buffer_NAD+_3-AB_0.1 M NaOH
AUC = 295
buffer_NAD+_ 98-4_0.1 M NaOH
AUC = 250
buffer_NAD+_ 08-9_0.1 M NaOH
rPtxs1_NAD+_3-AB_MQ
AUC = 572
rPtxs1_NAD+_ 98-4_MQ
AUC = 1274
rPtxs1_NAD+_ 08-9_MQ
AUC = 505
rPtxS1_NAD+_3-AB_0.1 M NaOH
AUC = 1189
rPtxS1_NAD+_ 98-4_0.1 M NaOH
AUC = 2464
rPtxS1_NAD+_ 08-9_0.1 M NaOH
AUC = 596
Absorbance (260 nm)
Time (min)

Figure S10.

Figure S10. HPLC-based analysis of high pH-induced hydrolysis of BaAD, IiaAD and IqaAD – HPLC chromatograms. First, NAD+ was incubated with the BaAD, IiaAD and IqaAD precursors and rPtxS1 at room temperature for 72 hours to biocatalytically produce BaAD, IiaAD and IqaAD. Subsequently, the reactions were incubated at room temperature for 72 hours in MQ-water or in 0.1 M NaOH. Each reaction was injected twice to the HPLC system. The peak profiles of the HPLC chromatograms contain the area under curve (AUC) values if the HPLC system detected a BaAD, IiaAD or IqaAD peak above the baseline. Refer to the peak purity data in Fig. S11. The reported AUC values refer to the average AUC values of the two independent HPLC injections.

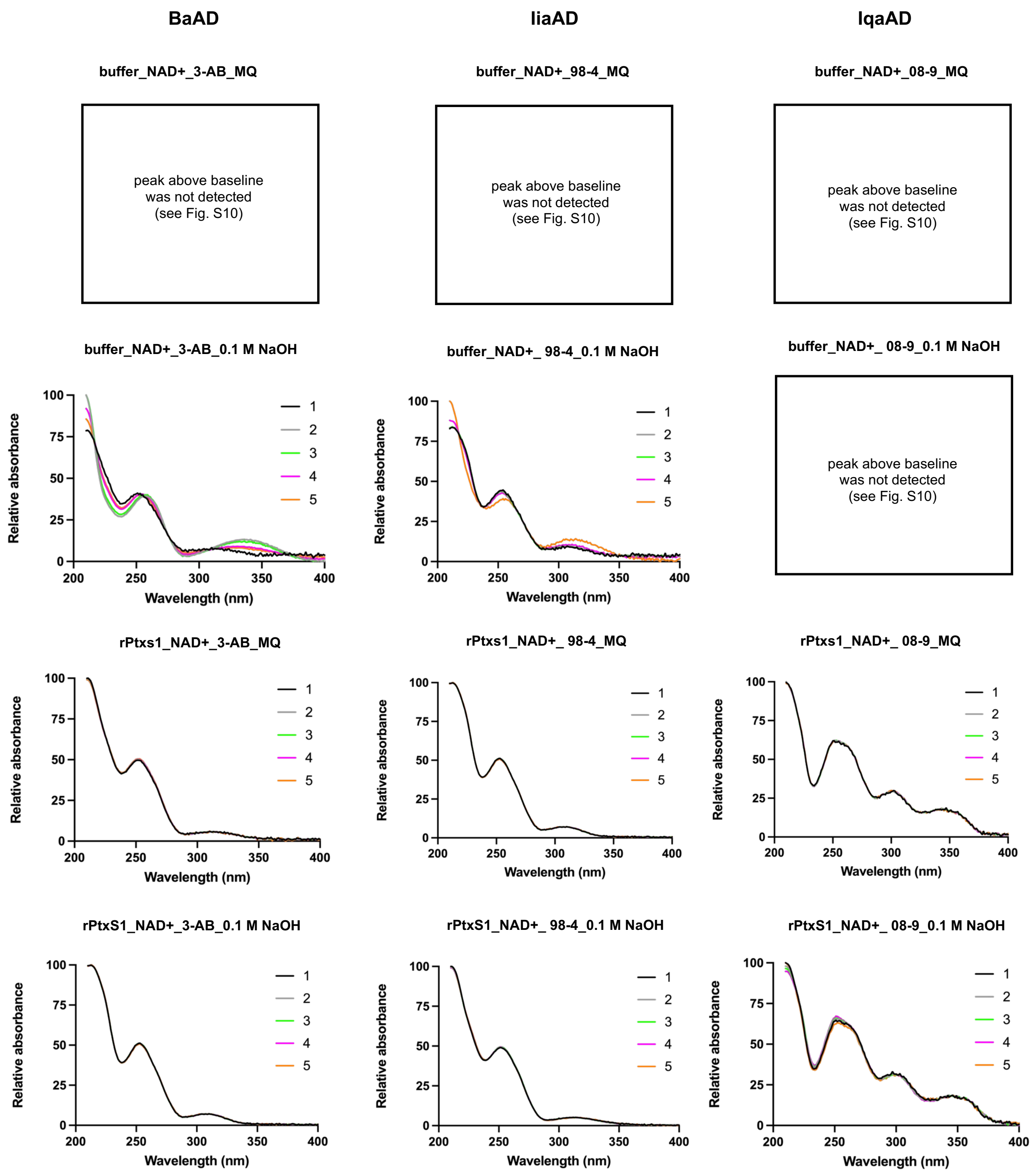
BaAD
IiaAD
IqaAD
buffer_NAD+_3-AB_MQ
buffer_NAD+_98-4_MQ
buffer_NAD+_08-9_MQ
peak above baseline
was not detected
(see Fig. S10)
buffer_NAD+_3-AB_0.1 M NaOH
buffer_NAD+_ 98-4_0.1 M NaOH
buffer_NAD+_ 08-9_0.1 M NaOH
rPtxs1_NAD+_3-AB_MQ
rPtxs1_NAD+_ 98-4_MQ
rPtxs1_NAD+_ 08-9_MQ
rPtxS1_NAD+_3-AB_0.1 M NaOH
rPtxS1_NAD+_ 98-4_0.1 M NaOH
rPtxS1_NAD+_ 08-9_0.1 M NaOH
Relative absorbance
Wavelength (nm)
1
2
3
4
5

Figure S11.

Figure S11. HPLC-based analysis of high pH-induced hydrolysis of BaAD, IiaAD and IqaAD – HPLC peak purity UV spectra. These peak purity UV spectra refer to the peaks as detected by the HPLC system in Fig. S10 singlet injections. Five measurements were executed for each detected peak at different locations of the peak once at the highest point, and two times from both sides of the highest point (see example of these locations in Fig. 5B).

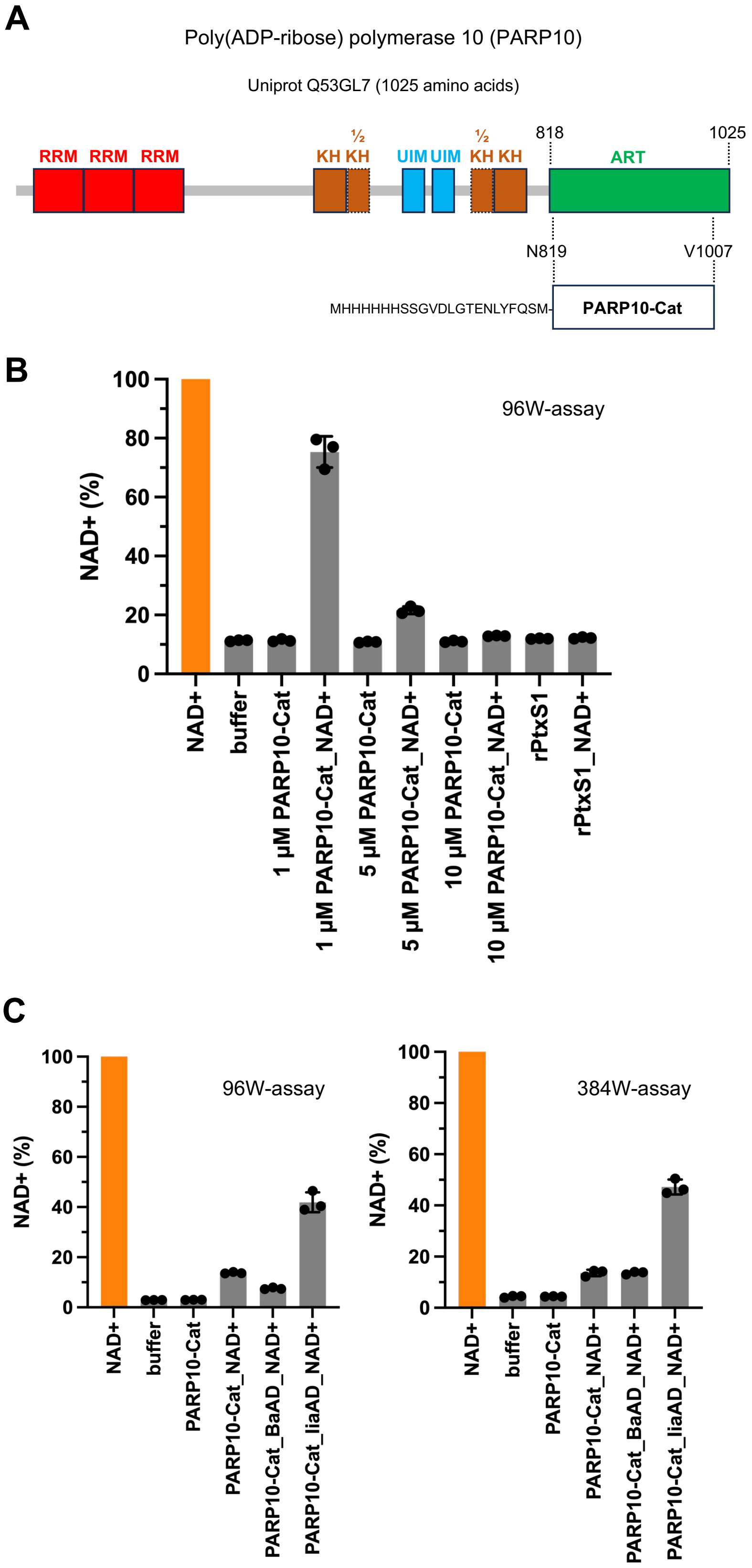
A
Poly(ADP-ribose) polymerase 10 (PARP10)
Uniprot Q53GL7 (1025 amino acids)
RRM RRM RRM
½ KH KH
UIM UIM
½ KH KH
818
1025
ART
N819
V1007
MHHHHHHSSGVDLGTENLYFQSM-
PARP10-Cat
B
96W-assay
NAD+ (%)
NAD+
buffer
1 µM PARP10-Cat
1 µM PARP10-Cat_NAD+
5 µM PARP10-Cat
5 µM PARP10-Cat_NAD+
10 µM PARP10-Cat
10 µM PARP10-Cat_NAD+
rPtxS1
rPtxS1_NAD+
C
96W-assay
384W-assay
NAD+ (%)
NAD+
buffer
PARP10-Cat
PARP10-Cat_NAD+
PARP10-Cat_BaAD_NAD+
PARP10-Cat_liaAD_NAD+

Figure S12.

Figure S12. Effect of BaAD and IiaAD on the NAD+ consumption activity of human PARP10. A) Schematic description of the HIS-tagged catalytic domain construct of PARP10 (PARP10-Cat), which we expressed in *E. coli* and purified with immobilized metal affinity and size exclusion chromatography techniques. The domain annotation not drawn on scale is based on Suskiewicz et al 2023 (https://doi.org/10.1093/nar/gkad514). RRM, RNA recognition motif; KH, K homology domain; UIM, ubiquitin-interacting motif, ART, ADP-ribosyltransferase domain. **B)** *In vitro* NAD+ consumption activity of PARP10-Cat and rPtxS1 (positive control, 2.5 µM) as measured with a 96-well fluorometric assay. NAD+ was used at 10 µM and it was chemically converted at the end of the 120 min consumption assay into a fluorescent molecule. Values are given as mean ± SD percentages of the NAD+ control (n=3, 1 representative experiment). **C)** BaAD and IiaAD (100 µM) were incubated with 4 µM PARP10-Cat for 30 min prior to addition of NAD+ (10 µM). NAD+ was chemically converted at the end of the 120 min consumption assay into a fluorescent molecule. The assay was run in two plate formats. Values are given as mean ± SD percentages of the NAD+ control (n=3, 1 representative experiment). IqaAD was not analyzed in this fluorometric assay due to its inherent fluorescence (see Fig. S8).

A

Compound	Docking score	Binding free energy before MD simulation by MM/GBSA (ΔG, kcal/mol)	Average binding free energy after 500 ns MD simulation by MM/GBSA +/- SD (ΔG, kcal/mol)
3-AB	-6.72*	-48.22*/-45.57**	-42.61 +/- 3.46
BaAD	-14.15	-74,04	-93.99 +/- 9.09
IiaAD	-14.71	-60.33	-90.75 +/- 8.66
IqaAD	-12.79	-68.60	-89.69 +/- 3.46

*from original co-crystallised 3-AB pose (PDB 6FXI)
**redocked 3-AB pose (PDB 6FXI)

B

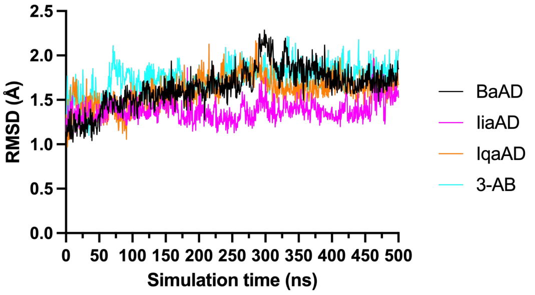

C

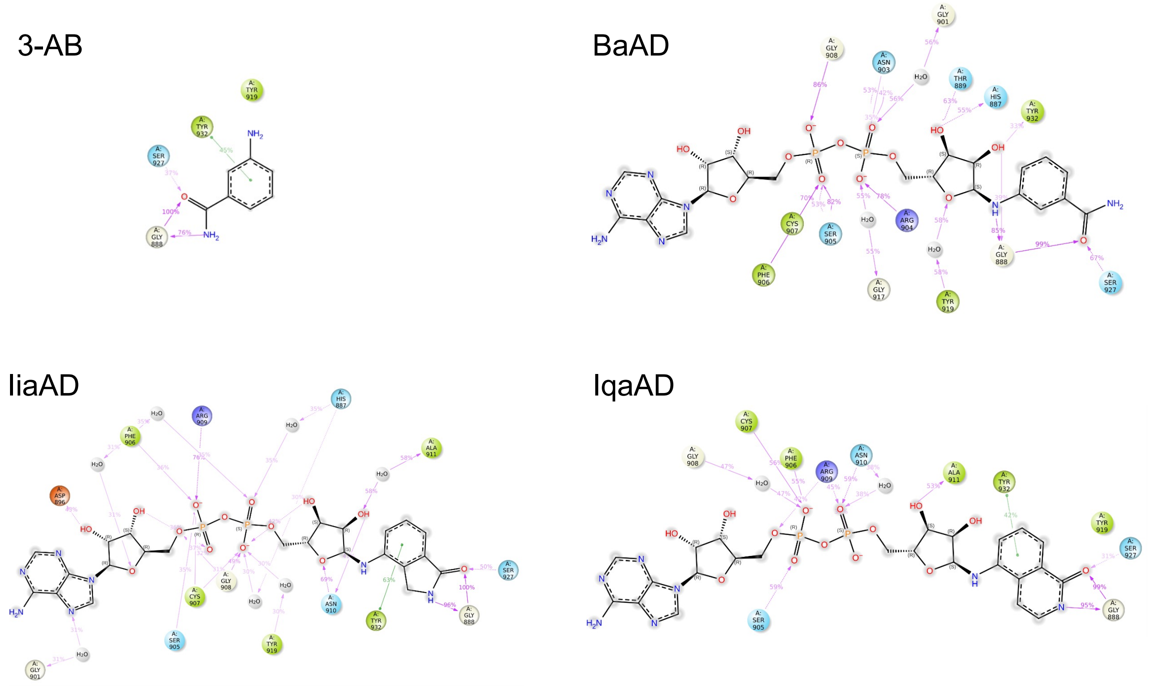

Figure S13.

Figure S13. Modeling of nucleotide binding to PARP10. A) Docking scores and Prime/MM-GBSA binding free energy (ΔG bind) values of different nucleotides (3-AB, BaAD, IiaAD and IqaAD) at the PARP10 catalytic fragment prior and after molecular dynamic (MD) simulations. The 3-AB bound crystal structure of PARP10 catalytic fragment (N819-V1009, Uniprot Q53GL7) was used as the modeling template (PDB 6FXI). **B)** The root mean square deviation (RMSD) of the PARP10 catalytic fragment during 500 ns MD simulations with different nucleotides. PARP10-Cat is more stable with IiaAD as compared with BaAD, IqaAD or 3-AB. The data reinforces the biochemical observation of more potent inhibitory effect of IiaAD on PARP10-Cat NAD+ consumption activity as compared with BaAD (see Fig. S12). **C)** Ligand-receptor interaction diagrams showing interactions of PARP10 catalytic fragment with 3-AB, BaAD, IiaAD and IqaAD over the course of a 500 ns MD simulation (% denotes the duration of an interaction from the total simulation time). Only interactions that lasted for more than 30 % of the simulation time are considered.
