# Supplementary material for: ADP-ribosyltransferase-based biocatalysis of nonhydrolyzable NAD+ analogs
Source: J Biol Chem. 2024 Dec 18;301(1):108106. doi: 10.1016/j.jbc.2024.108106 (PMC11786771; doi:10.1016/j.jbc.2024.108106)
Supplement: Supplementary Table S1 [file mmc3.docx]

**Table S1. Prediction of 3-AB analogs by *in silico* molecular orbital theorem assisted modeling exercise.** The modeling was executed to identify 3-AB analogs, which would have the capacity to insert into the nicotinamide pocket of PtxS1, and also would have the amino group-associated nucleophilic power to attack the PtxS1-formed oxocarbenium cation. Key characteristics of 3-AB and its analogs selected for biochemical experimentation are shown. The 3-AB analogs highlighted in blue yielded products by PtxS1-mediated catalysis.

|  |  |  |  |  |  |  |  |
| --- | --- | --- | --- | --- | --- | --- | --- |
| **Compound CAS number (internal name)** | **MW^a^** | **SASA^b^** | **Partition coefficient (LogP)^c^** | **Phase score^d^** | **HOMO**  **(kcal/mol)^e^** | **LUMO**  **(kcal/mol)^f^** | **Gap Energy**  **(kcal/mol)^g^** |
|  |  |  |  |  |  |  |  |
| 3544-24-9  (3-AB) | 136.15 | 329.72 | -0.36 | NA | -133.17 | -20.05 | -113.12 |
| 849035-63-8  (F023616) | 199.27 | 411.00 | 0.72 | 2.24 | -133.21 | -17.70 | -115.51 |
| 99-03-6  (F001722) | 135.16 | 344.10 | 1.47 | 2.25 | -126.74 | -36.92 | -89.83 |
| 17481-27-5  (F091967) | 166.18 | 369.62 | -0.17 | 2.31 | -127.95 | -17.99 | -109.96 |
| 19694-10-1  (F068290) | 170.59 | 348.86 | 0.13 | 2.56 | -137.63 | -24.19 | -113.44 |
| 19406-86-1  (F068209) | 150.18 | 356.07 | -0.02 | 2.16 | -131.81 | -18.18 | -113.63 |
| 60524-14-3  (F076704) | 137.14 | 321.29 | -0.98 | 2.72 | -139.60 | -28.73 | -110.87 |
| 6837-99-6  (BD19089) | 192.26 | 470.00 | 1.71 | 2.24 | -125.59 | -19.30 | -106.28 |
| 93117-08-9  (BD102168) | 160.18 | 348.21 | 0.40 | 2.25 | -119.20 | -21.44 | -97.76 |
| 871673-24-4  (HTS046169) | 176.22 | 418.33 | 1.20 | 2.30 | -125.81 | -19.72 | -106.09 |
| 3682-14-2  (BD6187) | 177.16 | 353.55 | -0.28 | 2.20 | -134.32 | -29.96 | -104.36 |
| 176032-78-3  (BD79018) | 160.18 | 365.74 | 0.86 | 2.25 | -135.44 | -19.52 | -115.91 |
| 366452-98-4  (BD94140) | 148.16 | 337.51 | 0.05 | 2.18 | -127.12 | -18.99 | -108.12 |
| 102562-86-7  (F227901) | 150.18 | 359.44 | -0.70 | 2.20 | -153.34 | -23.90 | -129.44 |
| 22961-58-6  (BD171615) | 166.17 | 369.78 | -0.12 | 2.31 | -123.78 | -14.65 | -109.13 |
|  |  |  |  |  |  |  |  |
| ^a^ molecular weight | | | | | | | |
| ^b^ solvent accessible surface area | | | | | | | |
| ^c^ predicted octanol/water partition coefficient values | | | | | | | |
| ^d^ phase score | | | | | | | |
| ^e^ highest occupied molecular orbital | | | | | | | |
| ^f^ lowest unoccupied molecular orbital | | | | | | | |
| ^g^ gap energy | | | | | | | |
|  | | | | | | | |
